# Supplementary material for: Activation of the Anaphase Promoting Complex Reverses Multiple Drug Resistant Cancer in a Canine Model of Multiple Drug Resistant Lymphoma
Source: Cancers (Basel). 2022 Aug 30;14(17):4215. doi: 10.3390/cancers14174215 (PMC9454423; doi:10.3390/cancers14174215)
Supplement: Supplementary file 1 [file cancers-14-04215-s001.zip › cancers-1873650-supplementary.pdf]

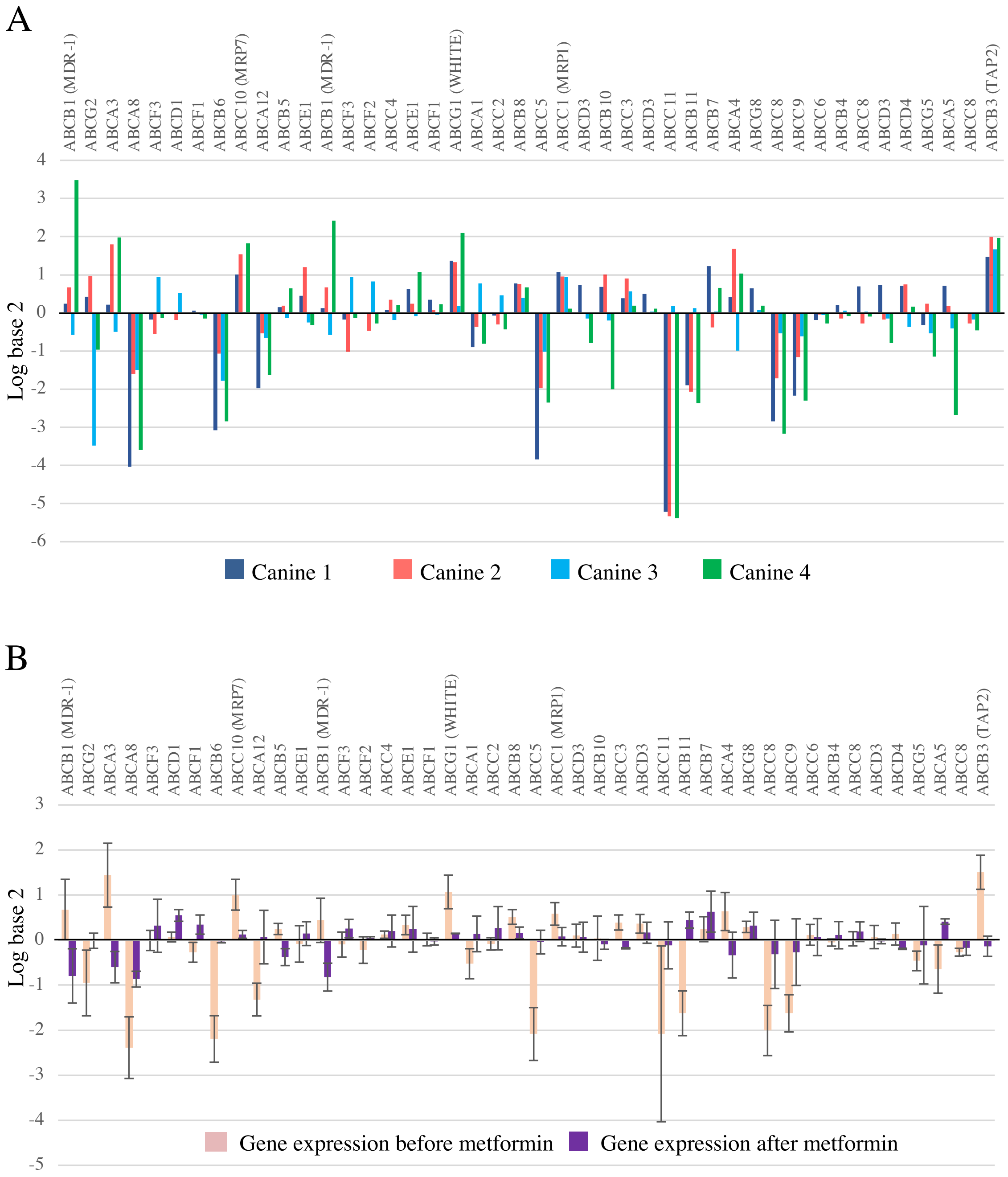
A
ABCB1 (MDR-1)
ABCG2
ABCA3
ABCA8
ABCF3
ABCD1
ABCF1
ABCB6
ABCC10 (MRP7)
ABCA12
ABCB5
ABCE1
ABCB1 (MDR-1)
ABCF3
ABCF2
ABCC4
ABCE1
ABCF1
ABCG1 (WHITE)
ABCA1
ABCC2
ABCB8
ABCC5
ABCC1 (MRP1)
ABCD3
ABCB10
ABCC3
ABCD3
ABCC11
ABCB11
ABCB7
ABCA4
ABCG8
ABCC8
ABCC9
ABCC6
ABCB4
ABCC8
ABCD3
ABCD4
ABCG5
ABCA5
ABCC8
ABCB3 (TAP2)
4
3
2
1
0
-1
-2
-3
-4
-5
-6
Log base 2
Canine 1
Canine 2
Canine 3
Canine 4
B
ABCB1 (MDR-1)
ABCG2
ABCA3
ABCA8
ABCF3
ABCD1
ABCF1
ABCB6
ABCC10 (MRP7)
ABCA12
ABCB5
ABCE1
ABCB1 (MDR-1)
ABCF3
ABCF2
ABCC4
ABCE1
ABCF1
ABCG1 (WHITE)
ABCA1
ABCC2
ABCB8
ABCC5
ABCC1 (MRP1)
ABCD3
ABCB10
ABCC3
ABCD3
ABCC11
ABCB11
ABCB7
ABCA4
ABCG8
ABCC8
ABCC9
ABCC6
ABCB4
ABCC8
ABCD3
ABCD4
ABCG5
ABCA5
ABCC8
ABCB3 (TAP2)
3
2
1
0
-1
-2
-3
-4
-5
Log base 2
Gene expression before metformin
Gene expression after metformin

Supplemental Figure S1. ABC transporters in general do not appear to play a critical role in the development of canine MDR lymphoma. A. Differential gene expression of the family of ABC transporters on the canine microarray was determined for each MDR canine sample. **B.** ABC transporter gene expression changes were averaged for the 2 canines (2 and 4) that were treated with metformin and analyzed by microarray. The light purple bars represent MDR tumor before metformin compared to controls. The bars in dark purple represent MDR tumors after metformin treatment compared with before treatment. The SEM was plotted.

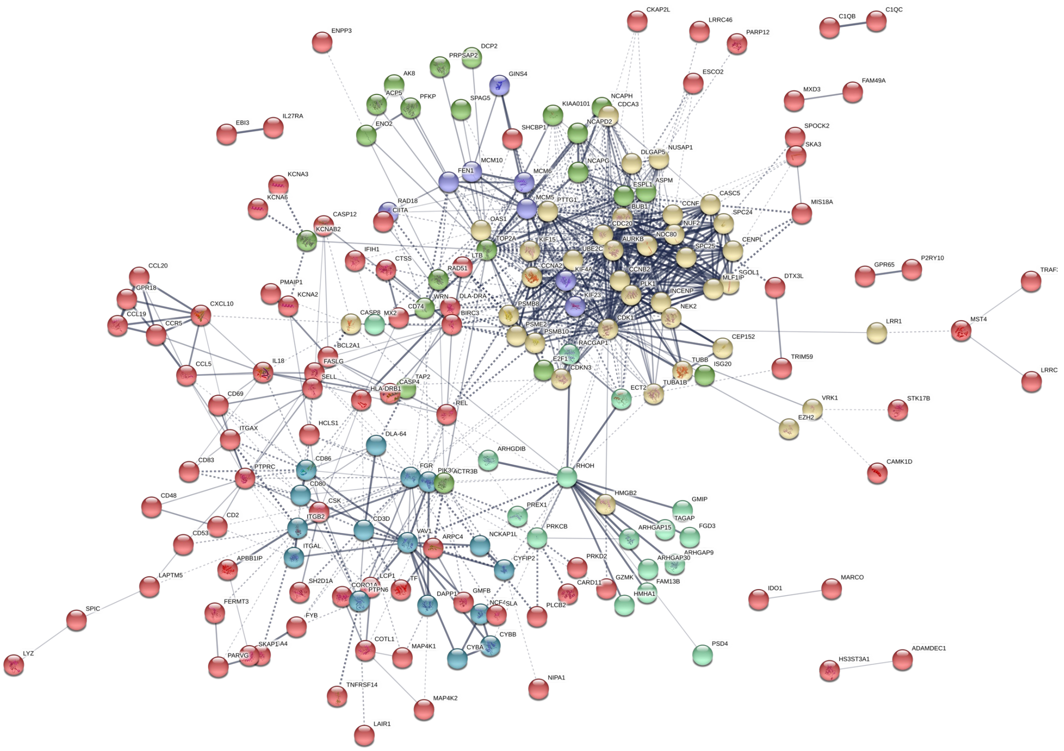

Supplemental Figure S2. 186 genes form the 290 gene set are predicted to form a highly interconnected network based on STRING. STRING analysis of the 290 common overexpressed genes within the tumors of MDR canines (Fig. 2A) reveals a highly interconnected 186 gene set. A thicker connecting edge indicates higher confidence in the predicted interaction. The genes were grouped into 6 clusters, as shown by the different colored nodes. The yellow nodes are highly clustered and largely define genes involved in mitotic progression (see Supplemental Table S4). The green and purple nodes, also tightly connected to the yellow nodes, are primarily involved in chromosome maintenance and DNA repair. A significant number of these genes encode proteins known to be targeted by the Anaphase Promoting Complex (marked by an * in Supplemental Table S4). The majority of these genes are elevated in a variety of human cancer types.

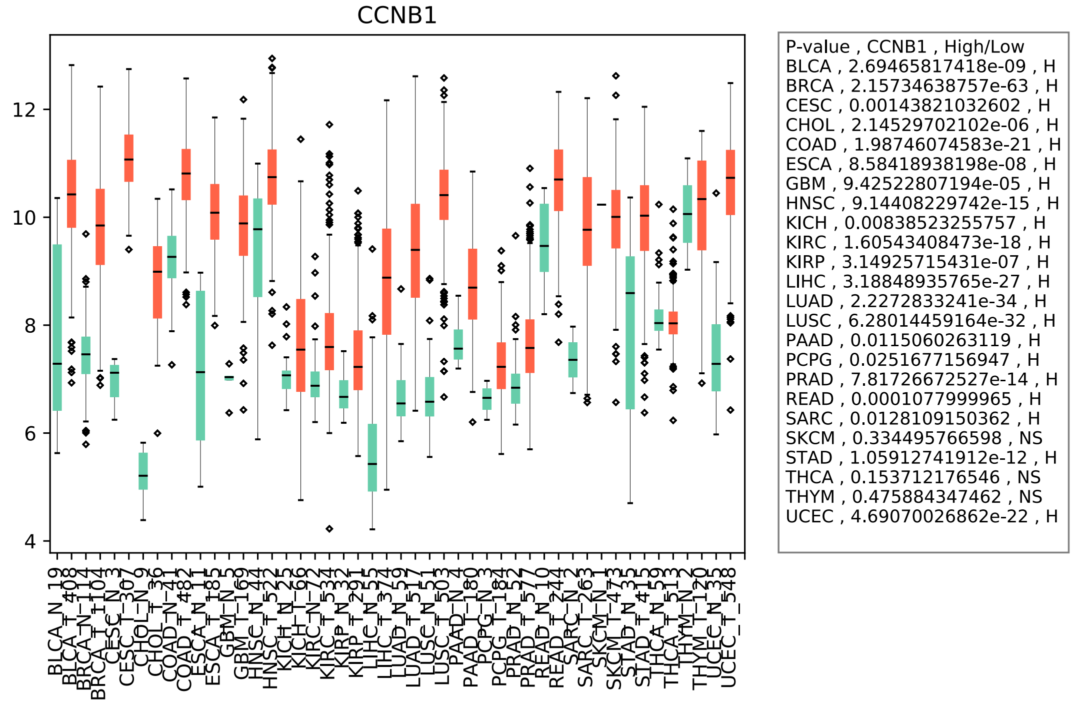

Supplemental Figure S3. Expression scores for CCNB1 within 24 different types of cancer and normal tissue. Using the Cancer Genome Atlas (TCGA) (https://portal.gdc.cancer.gov/) database, we determined if expression of an APC substrate gene (CCNB1) in cancer patients is differentially regulated between normal and the tumor tissues. The numbers along the x-axis denote the number of patient samples in each cancer type. Statistical significance of the difference in expression between the normal and tumor samples are depicted for each cancer type. NS denotes not significant. The abbreviation of each cancer along the y-axis is represented as described in the TCGA portal.

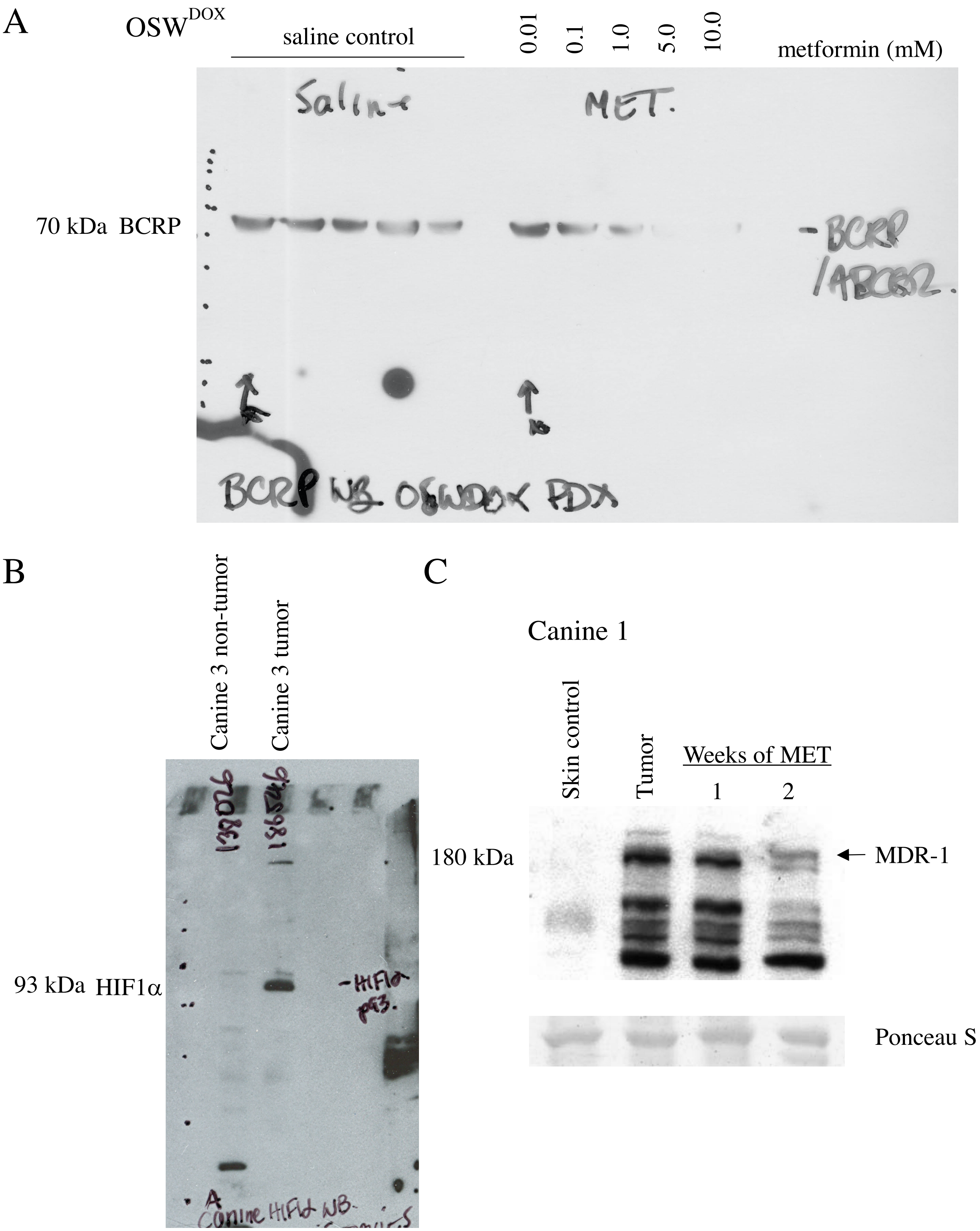
A
OSW^{DOX}
saline control
0.01
0.1
1.0
5.0
10.0
metformin (mM)
70 kDa BCRP
B
Canine 3 non-tumor
Canine 3 tumor
93 kDa HIF1α
C
Canine 1
Skin control
Tumor
Weeks of MET
1
2
180 kDa
MDR-1
Ponceau S

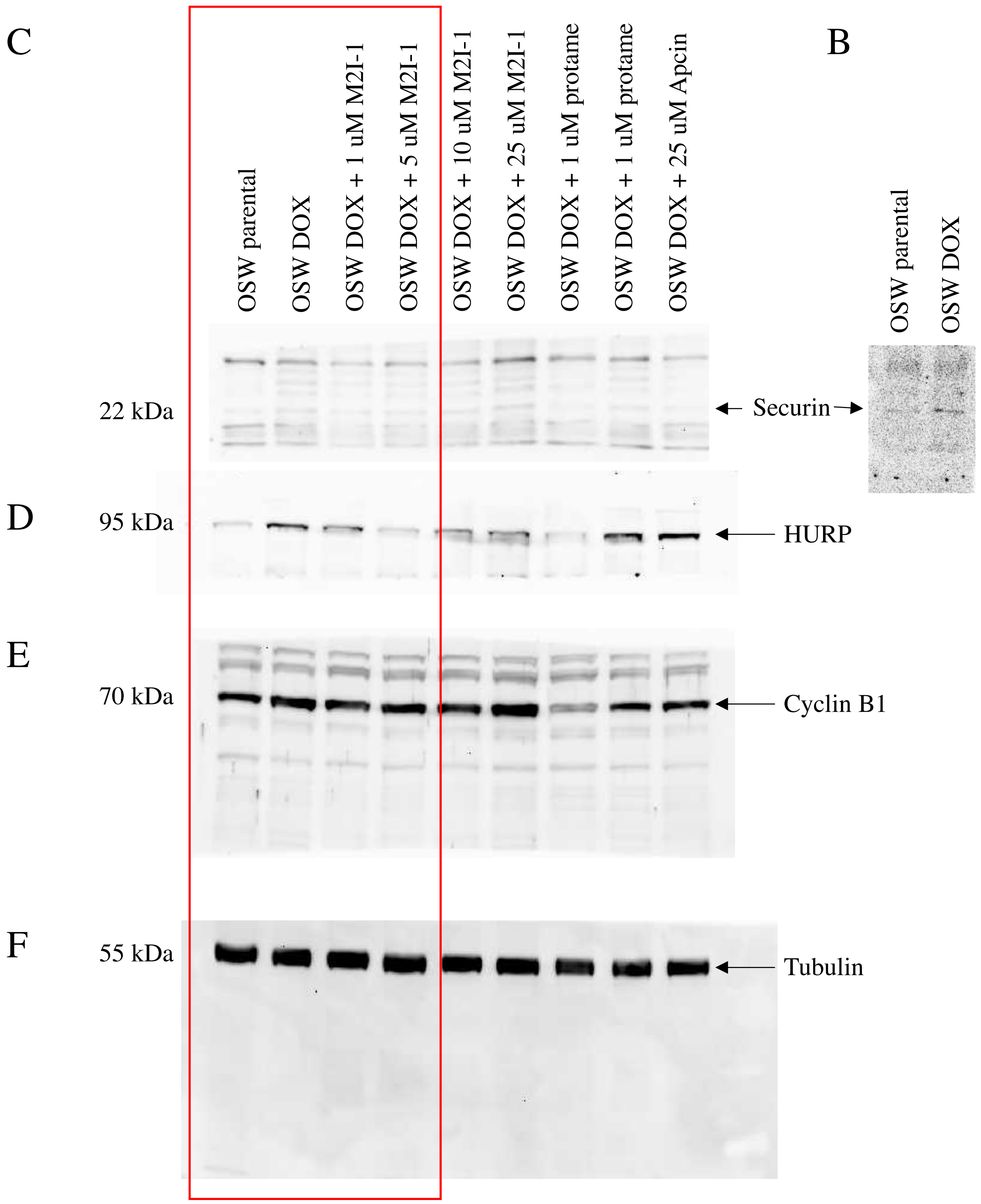
C
OSW parental
OSW DOX
OSW DOX + 1 uM M2I-1
OSW DOX + 5 uM M2I-1
OSW DOX + 10 uM M2I-1
OSW DOX + 25 uM M2I-1
OSW DOX + 1 uM protame
OSW DOX + 1 uM protame
OSW DOX + 25 uM Apcin
B
OSW parental
OSW DOX
22 kDa
Securin
D
95 kDa
HURP
E
70 kDa
Cyclin B1
F
55 kDa
Tubulin

Supplemental Figure S4. Original blots. These are representative blots showing the full range of bands observed using the different antibodies assessed in this study. **A.** A complete western blot showing BCRP protein bands. See Figures 1C and 5A. **B**. A complete western blot showing signal using HIF1α antibodies. See Figures 1C and 5A. **C**. A complete blot showing signal for antibodies against MDR-1. See Figures 1 and 5A. **D**. and **E**. Typical Securin western blot results. A band at 22 kDa was imaged for Securin. See Figures 5C and 5E. **F**. A blot showing the bands observed using antibodies against HURP. See Figures 5C and 5E. **G**. A western blot showing the results of using antibodies against Cyclin B1. See Figure 5C. **H**. Western blot showing results using antibodies against Tubulin. See Figures 1B, 1C, 5C and 5E.

Supplemental Table S1. Lists of genes upregulated >3 fold in the 4 MDR canines. All genes differentially expressed >3 fold for the tumor samples, compared to control skin samples, were retreived from the microarrays. Venn analyses identified a common set of 290 genes overexpressed in all 4 MDR canines. STRING analyses identified a set of 186 genes within the 290 gene set that were highly connected.

Supplemental Table S2. Cytoscape node list for the 146 gene set (see Fig. 2B). The 290 gene set was analyzed using the Cytoscape online tool. 13 interconnected nodes, composed of 146 genes, were identified that defined pathways involved in DNA repair and cell cycle progression through mitosis.

Supplemental Table S3. Cytoscape network pathway enrichment for 146 gene set (see Fig. 2B). Network, Biological and Cell Component pathways were identified from the 146 gene network indicating the number of total genes in the complete gene set and the number found in our network. The enrichment was indicated by the p-value.

Supplemental Table S4. Gene clustering from the STRING analysis identifies a set of genes within the 186 gene set that defines progression through mitosis as a key regulator of MDR development. The clusters identified in Supplemental Fig. S2 were listed and categorized in terms of function based on review of the literature. Genes highlighted in yellow define genes involved in mitotic progression, while genes highlighted in green define genes involved in DNA replication and repair. * denotes APC substrates; ** denotes kinetochore and Spindle Assembly Checkpoint associated proteins; *** denotes components of the chromosome condensin complex.

Supplemental Table S5. Analyses of genes identified as responsive to clinical chemoresistance, remission, and relapse in canine 4. A table comparing genes overexpressed >3 fold in the chemoresistant tumor, down-regulated >2 fold upon remission, and >3 fold following relapse in canine 4, identified 27 genes in common. STRING interactions were identified for each comparison. As above, genes highlighted in yellow define genes involved in mitotic progression, while genes highlighted in green define genes involved in DNA replication and repair. * denotes APC substrates; ** denotes kinetochore and Spindle Assembly Checkpoint associated proteins; *** denotes components of the chromosome condensin complex.

Supplemental Table S1: Genes upregulated in the 4 canines over 3 fold revealed a common 290 gene set.

Canine 2-Tumor vs Control-875 genes	Canine 4-Tumor vs control-1254 genes	Canine 1-Tumor vs control-1276 genes	Canine 3-Tumor vs control-1365 genes	Venn 290 common gene set	STRING 186 common
CXCL13	CD52	AK8	LOC612122	PFKP	PFKP
TF	DNAH8	AK8	IGHAC	NCF4	NCF4
MARCO	LOC607937	CXCL13	BEX5	HS3ST3A1	HS3ST3A1
CCL19	IL7R	LOC478056	PCDHA5	KCNA6	KCNA6
LOC612122	GZMK	NCF4	SAMSN1	MLF1IP	MLF1IP
LOC612180	LOC612122	IGHAC	TF	FGR	FGR
CD5L	CD8A	BEX5	SELL	SEMA4D	PRKCB
DNASE1L3	LOC488683	SELL	LOC102155211	C20H19orf66	SELL
APOC1	GGT1	LOC612180	CCL19	PRKCB	CCL19
SFRP2	LOC612180	LOC102155211	ISG20	SELL	LAIR1
LOC607937	ZNF683	CXCL13	CASP12	CPLX3	LRRC8D
CCL20	GPR171	PCP4L1	LOC612180	CCL19	CDK1
WFDC2	FYB	FYB	ITGB2	DLA-DMB	TUBB
TNFSF11	CD40LG	LOC612122	CASP12	LAIR1	RACGAP1
CD86	SH2D1A	CD52	CXCL10	LRRC8D	TF
TF	TNFSF8	LOC100855883	HAAO	CDK1	FYB
CXCL10	CD226	CPLX3	DLA-DOB	TUBB	RAD18
POSTN	GIMAP7	LOC607937	AK8	RARRES3	RHOH
GZMK	CD96	PHEX	SFRP2	RACGAP1	FERMT3
MPO	IL12RB2	SCML2	TNFSF8	DUSP5	DAPP1
PCDHA5	TRAT1	TRAF3IP3	OASL	TF	CD53
CAMP	CD3E	GIMAP2	CYBB	FYB	CIITA
LOC100686390	CD80	MIS18A	TRAF3IP3	RAD18	GMFB
C1QC	LEF1	SH2D1A	CD5L	RHOH	ARHGAP15
SH2D1A	SLAMF1	RBM44	PMAIP1	IKZF3	IL27RA
CD52	SYTL3	LOC612553	LOC608687	FERMT3	TAGAP
KMO	CCL19	BUB1B	ELL3	DAPP1	AURKB
CR2	CD28	KMO	CASP4	CD53	OAS1
RGS10	LCK	CYBB	PLEKHA2	CIITA	SLA
POSTN	CASP12	SLC38A11	CD69	LYSMD2	CENPL

LOC612553	CD160	CDCA3	ADAMDEC1	GMFB	KIF4A
LAIR1	LCK	GPR65	LOC100856041	ARHGAP15	LRR1
IDO1	GFI1	KIAA0101	RBM11	IL27RA	ENO2
CD80	CASP12	ELL3	PTCHD2	GFI1	FAM49A
TIMD4	CD27	CCNB2	HLA-DRB1	FAIM3	CD2
AMICA1	CCL5	DNTT	LRRC46	TAGAP	MCM10
CD69	IGHAC	TNFSF8	DLA-DQB1	AURKB	CD83
CCL5	CD8B	MX2	CIART	TFEC	IL18
ASPM	ICOS	AGER	FXYD2	DLA88	MCM5
PTTG1	CTSW	RHOH	CD1A8	OAS1	ITGB2
VCAM1	ADAMDEC1	LRRC46	CCR6	SLA	PSMB8
TFPI2	AMICA1	PLEKHA2	CYBB	CENPL	PIK3CD
CBD139	GIMAP2	PTTG1	LIMD2	ND6	NDC80
SNAP91	RGS1	RBM11	NDC80	KIF4A	GPR18
CD2	IDO1	GPR15	THEMIS2	LIMD2	CCNA2
LOC100685470	CLIC6	LOC100856041	WFDC2	LRR1	SPIC
TNFSF8	LOC608687	RACGAP1	LOC100685195	ENO2	NUF2
IL18	CD69	RARRES3	MIS18A	RAB11FIP4	CD86
ND6	PSTPIP1	CD69	LY86	FAM49A	PTTG1
CCNB2	AMICA1	PSPH	CORO1A	CD2	BCL2A1
CLEC12A	ND6	ESPL1	KMO	MCM10	ECT2
NCAPG	CD6	LY86	HAVCR1	CD83	KIF23
MILR1	IL2	MLF1IP	HSH2D	IL18	PTPRC
RBP5	CD80	BUB1	OAS1	SMCO4	ARHGDIB
CCL4	CTLA4	CCL19	DLA-DQB1	TRAIP	PARVG
SLC38A11	BEX5	SHCBP1	LOC606863	LOC607281	IDO1
CCL2	CD2	MX1	DNASE1L3	MCM5	EBI3
LOC608687	CCR5	BLK	CSPG5	ITGB2	SPAG5
AMICA1	MARCO	GCA	CR2	GDPGP1	ACTR3B
EBI3	GRIP2	LOC100685470	BLK	PSMB8	PARP12
CORO1A	ISG20	ZG16B	EFCAB3	ABRACL	HMHA1
FABP2	TAGAP	ASPM	USP18	PIK3CD	ACP5
LIMD2	LOC612106	CDKN3	DLA-DQA1	NDC80	AK8

SLC14A1	GIMAP8	HHAT	CCL20	GPR18	TAP2
CDK1	GIMAP4	KIAA0226L	RHOH	CCNA2	PRKD2
IFI6	GBP1	CCL17	LOC611500	LOC481744	CCNB2
BUB1B	LOC488248	NCAPG	FGR	SPIC	KCNA3
CD53	DNASE1L3	RAB11FIP4	PADI4	DLA-DOB	PSD4
FYB	ZAP70	CDC20	FAM118A	NUF2	CD74
IL18BP	RUNX2	RAB11FIP4	NCF2	THEMIS2	NCKAP1L
LAPTM5	BST2	MYBL2	KIAA0101	CD86	CYBB
NCF4	SIT1	KIF23	LOC100685470	PTTG1	PMAIP1
FUT8	LOC611813	CBD139	ACP5	STMN1	ARPC4
VWA5A	CCL4	DLA-DOB	CD19	BCL2A1	TNFRSF14
NDC80	RGS10	DLGAP5	MX2	EBP	P2RY10
CYBB	DOK2	EXO1	PLCG2	PARP8	PTPN6
ADORA3	RARRES3	C4BPA	BIRC3	ECT2	ITGAX
TRAF3IP3	PREX1	BANK1	GIMAP2	FMNL1	DLGAP5
TTK	LAPTM5	BANK1	PTPN6	KIF23	ESCO2
BFSP2	SLA	SKA3	CIITA	PTPRC	CXCL10
HS3ST3A1	EMB	UBE2C	HHAT	ARHGDIB	CKAP2L
RARRES1	FAM65B	NCAPH	IFIH1	PARVG	CCL5
BEX5	KCNA6	DEPDC1B	MMP9	IDO1	PRPSAP2
LOC490399	SCML4	PMM2	GZMK	EBI3	MAP4K2
CD80	RHOH	BATF	LRMP	C27H12orf35	TRAF3IP3
FASLG	TRAF3IP3	GIMAP4	AK8	SPAG5	LYZ
GPR171	DLA-DOB	HSH2D	CD86	ACTR3B	LCP1
SLAMF1	CLEC9A	HAVCR1	TF	PCDHA5	FGD3
LRMP	RUNX2	ARHGAP11A	DLA-DMB	PARP12	CCNF
CASC5	GBP1	EAF2	OAZ3	HMHA1	LRRC46
CD180	PIK3CD	IL7R	PHEX	ACP5	IFIH1
CCL17	EMR2	NDC80	BIN2	TRIM34	DLA-64
ELOVL2	KCNA3	GFI1	ATP6V0D2	AK8	PREX1
CDKN3	SLA2	CDC45	HTR1D	TAP2	MARCO
ESPL1	GCNT4	LOC475113	C20H19orf66	PRKD2	PSME2
CDCA3	LOC100685470	AURKB	LOC610699	CCNB2	CASP4

CCNB3	CD53	PTPRJ	HS3ST3A1	GIMAP4	KIAA0101
CD82	INPP4B	SLC9B1	CD72	KCNA3	FAM13B
SERPINE2	ICOS	TTK	PIK3CD	GBP1	CYFIP2
ALOX15	CXCR4	PLK4	CD74	PSD4	ENPP3
SPAG5	KCNA2	CXCL10	NCF1	BIN2	NEK2
NUF2	BIN2	SPC24	ESCO2	CD74	ITGAL
GPR65	LOC490356	PRKCB	ZMAT1	LOC612180	SPC24
BTK	DTHD1	CD1A8	PFKL	TNFSF8	ESPL1
UBE2C	IL18RAP	FABP2	CPLX3	NCKAP1L	CYBA
IFGGB1	DENND2D	CASC5	CTSZ	SWSAP1	C1QC
CD33	LCP2	CCNB3	WDFY4	CYBB	PLCB2
OASL	DLA-DQB1	ECT2	RPS6KA1	PMAIP1	SH2D1A
DLA-DQB1	TXNDC5	CCL20	LOC490917	LRMP	ARHGAP30
C1QB	CAMP	HAAO	LOC607055	ARPC4	ADAMDEC1
NEUROD6	PMAIP1	KAZALD1	PARP9	TNFRSF14	CARD11
POLE2	LOC490595	MIS18A	RARRES2	P2RY10	PLK1
ADAMDEC1	CFP	NCF2	IFGGB1	PTPN6	MIS18A
DLA-DQB1	CD86	DEPDC1	DERL3	WFDC2	KCNA2
CPLX3	DLA-DQB1	LOC608687	FAM122B	ITGAX	EZH2
TOP2A	GPR65	MSANTD3	CD79B	DLA-DQB1	DTX3L
RAB11FIP4	CXCR3	NEK2	DNASE2	DLGAP5	E2F1
LOC611500	CMA1	BST2	CLEC12A	PLEKHA2	CASP8
CTSG	IGJ	POLR3F	MELK	ESCO2	GZMK
ENPP3	LOC609038	NUSAP1	PTPN22	CXCL10	TUBA1B
LOC490356	CARHSP1	MCM10	TLR1	CKAP2L	MAP4K1
CCL8	CYTIP	CCNA2	PTTG1	CCL5	CSK
SKA3	IFGGB1	SLC51B	SLC5A3	PRPSAP2	CTSS
DLGAP5	CASP3	UNC119	MYBL2	MAP4K2	SGOL1
KIF23	CRTAM	SAP25	BCL11A	TRAF3IP3	GMIP
NUSAP1	CASP4	HELLS	DLA-DRA	LYZ	LTB
LOC448801	LOC606863	TOP2A	PARP14	LCP1	CCL20
KIAA0101	LOC100684769	CHEK1	AIFM3	FGD3	CDC20
FHIT	CXCL10	LOC476070	PRKCB	CCNF	CD3D

A2M	PRKCB	NCAPG2	LTB	LRRC46	BUB1
NTS	SELL	MCM5	CASC5	IFIH1	HMGB2
CD72	BCL11B	POC1A	ARHGAP30	DLA-64	MX2
RAB11FIP4	FBXO5	ORC6	AURKB	PREX1	ITGA4
NEUROD6	CD7	MCCD1	FGD2	MARCO	SKA3
PHEX	BPIFB1	ATAD5	ARHGAP11A	ST3GAL5	NCAPH
CD1E	CTSG	CD180	LYSMD2	SDF2L1	FASLG
BCL2A1	RTN4RL1	GINS4	ARHGDIB	PSME2	GPR65
KNSTRN	LOC100856041	GPR171	IGSF9	CASP4	SHCBP1
CDC20	SEMA4A	RAD51	C6	KIAA0101	COTL1
LTB	REG3A	GBP1	SEMA4D	FABP2	SKAP1
TNFRSF14	EFHC2	SPC25	GRK6	FAM13B	ASPM
BATF	ALDOC	KIF4A	LOC488248	CYFIP2	ARHGAP9
TRIM22	HLA-DRB1	DNASE1L3	COTL1	LPCAT1	CDCA3
TLR8	TMEM127	APOC1	UBE2C	ENPP3	DLA-DRA
TLR1	CST7	CENPI	EPSTI1	NEK2	CD80
CD19	CXCR4	IDO1	FGR	ITGAL	UBE2C
GFI1	CMTM7	OAS1	CMTM7	SPC24	KCNAB2
SAMSN1	SLC9A3R1	LOC611500	FCRL1	DERL3	GINS4
MYBL2	SH2D2A	ORC1	FAIM3	ESPL1	CASC5
BUB1	CABP5	ITGB2	KIAA0226L	PTPRCAP	SPOCK2
CASP12	LOC484314	SKA3	KYNU	CYBA	CORO1A
GIMAP2	CCND3	TLR1	ARL5C	C1QC	TOP2A
SPIC	PTGDR	PTPN22	LAPTM5	PLCB2	FEN1
ARL4C	ST8SIA4	CDK1	GIMAP4	SH2D1A	NCAPD2
GPR18	CD33	IFIH1	RASGRP1	TRIM22	DCP2
TMEM176B	LRRC46	CR2	NCAPG	CD33	ISG20
CD40	FGD3	DAPP1	STMN1	DLA-DQA1	SPC25
PLK1	RASSF5	SPAG5	CD180	ARHGAP30	MXD3
CCR5	PTPN22	SNX10	CD83	ADAMDEC1	C1QB
TMEM150B	cOR52H7	ISG20	CD74	CARD11	KIF15
SHCBP1	TRIM34	SELP	TBC1D10C	PLK1	CAMK1D
CDH11	LOC100856122	GPR18	BUB1	IFGGB1	WRN

APOE	RAB11FIP4	CENPK	BUB1B	MIS18A	CD48
OPRK1	CIITA	NUF2	DNAH10	IKZF1	NIPA1
AOAH	IPCEF1	AOAH	DLA-DOA	KCNA2	REL
NEIL3	CCR7	HTR1D	TNFSF11	DOCK8	MST4
AK8	COTL1	RGS10	DOCK8	EZH2	HLA-DRB1
PLK4	DLA-DQA1	SGOL1	IFI44L	DNASE1L3	RAD51
RHOH	IFIT3	BIRC3	ESPL1	DTX3L	LAPTM5
IL18BP	BCL2A1	CASP8	CD2	GMCL1	MCM6
TLR7	ZFP36L2	OASL	SNAP91	KNSTRN	CCR5
SLPI	CCL20	LRMP	CYBA	E2F1	CEP152
HSH2D	LOC100683878	FANCM	CBD139	CASP8	CD69
SELL	PDE3B	CORO1A	BCL2A1	PLXNC1	TRIM59
TREML1	HMHA1	CYBA	ACTR3B	SNX10	APBB1IP
KPNA2	SKAP1	LOC475176	VAV1	GZMK	PSMB10
SEMA4A	PRKD2	TENM3	METTL4	TRIM37	CDKN3
CACNB4	ITGB2	GINS2	PPP1R3E	TUBA1B	CASP12
ISG15	RAB19	ENO2	NUSAP1	LOC100685470	NCAPG
IL7R	ABCB1	PFKL	TMEM130	MAP4K1	STK17B
TFEC	LOC611500	LOC606863	FASLG	CSK	VRK1
KIF4A	RAB27A	LOC607055	PLCB2	CTSS	VAV1
BIRC3	FAM65B	STMN1	DAPP1	SGOL1	NUSAP1
VRK1	TRIB2	AGPAT3	ND6	LOC483397	INCENP
CD83	BATF3	CEACAM1	ISG15	GMIP	HCLS1
SPC25	MMP12	CSPG5	DLA-12	UNC13D	BIRC3
VASH2	FAM155B	KNSTRN	HMHA1	LTB	
PAG1	EOMES	RHEBL1	CCR5	TLR8	
LOC482124	SLA	WNK4	ASPM	ACRBP	
DLA-DRA	KCNK5	E2F8	C4BPA	ARHGEF39	
MX1	MX2	XAF1	LYN	CCL20	
LYZ	GRK6	FAM72A	MCCD1	LOC100684769	
C1QA	LOC607055	DENND5B	CYFIP2	LOC607055	
RGS18	SPERT	CD53	LOC477558	CDC20	
CDC25C	TRIM22	BIN2	TRIM22	CD3D	

WDFY4	AIFM3	NCAPD2	GPR15	BUB1
TSHB	ARHGAP30	DLA-DMB	LRMP	AIF1
RAD51	RAP1GAP2	PAX6	LOC490399	CBD139
APOBEC3H	LIMD2	CD70	SIGLEC10	ZNF292
PDCD1LG2	FAIM3	CTSL3	CSK	OASL
CYBB	METTL21D	CCNF	TTK	LOC607937
PBK	STK17A	CA11	SWSAP1	HMGB2
ECT2	ITGAL	FAM83D	STK17B	BATF
AIF1	OASL	MELK	FAM72A	TMEM154
LOC476070	LTB	CFTR	ENO2	MX2
TRAIP	DLA-DRA	ARL5C	OAS3	ITGA4
GRIN2B	CD5L	SEMA4D	DDX58	RILPL2
UNC119	RAB11FIP4	CDC25C	NFKB2	SKA3
CENPF	GBP6	EMR3	CASP10	NCAPH
PLCB2	CELF4	NCKAP1L	MX1	FASLG
TRIM22	DLA-12	USH1C	LOC100684769	GIMAP2
TMPRSS7	SRGN	IFGGB1	CASP8	LOC611500
DEPDC1	GRAP2	NEIL3	ADAM23	LOC100856041
MIS18BP1	BATF	DTL	COTL1	GPR65
ARHGAP15	DLA-DMB	HPSE	AURKA	SHCBP1
FAM83D	KIAA1598	CENPA	LAIR1	COTL1
CD79B	SH3BP5	FUT8	DLA-DMA	PML
TROAP	TRIM22	DNASE2	MIS18A	SKAP1
HMHA1	ZMAT1	PBK	PML	ASPM
DDX58	SLC4A7	FERMT3	GALNT12	ARHGAP9
ACP5	HS3ST3A1	DCK	LOC102153030	CDCA3
HLA-DRB1	LCP2	DENND5B	CCNB2	DLA-DRA
PYY	MST4	PLCG2	IKZF3	FAM65B
MIS18A	FABP2	ESCO2	PRKD2	CD80
SPC24	LOC480600	ISG15	LOC483397	PHF6
CMTM7	LOC100684560	FAM118A	STX11	LOC608687
C18H7orf72	ETFB	CDC6	CCL5	CD5L
STK17B	PLEKHA2	LOC478994	RALGPS2	UBE2C

NR1H3	TAPBPL	USP18	ZDHHC14	KCNAB2
FGD3	COR2T18	ND6	OAS2	GINS4
ADAM23	MPO	HLA-DRB1	MLPH	LOC612122
TNFRSF14	CCND2	KCNA3	C31H21orf91	CASC5
CCDC63	KLRG1	CENPN	ZDHHC18	SPOCK2
CSK	ARL4C	GZMK	TLR7	TBC1D10C
PARVG	ETS1	BCL2L14	ARHGAP15	APOBEC3H
DLA-DMB	TBC1D10C	LRRC25	FAM65B	RBM11
DHCR24	APOC1	GPSM3	GPR65	SASH3
ARHGAP30	TARP	CENPF	HERC5	CORO1A
SBSPON	C4BPA	MARCO	C1QC	GPSM3
CRIP3	DLA88	POLD1	BTK	TOP2A
NCF2	LOC100855540	CHEK2	TOP2A	FEN1
RBM47	LOC609402	PARP14	GCA	ABAT
SLC1A2	SAP25	FOXM1	CCND2	NCAPD2
ABRACL	TRERF1	POLR3F	PTPRC	DCP2
IFIH1	CD3G	TRIM22	CTSH	ISG20
OOEP	F12	ANAPC1	TRIM22	SPC25
CFP	SASH3	IFNK	MOV10	MXD3
SPOCK2	AIF1	TOP2A	CDKN3	HSH2D
LRRC25	TRAF5	KIFC1	CDK1	SLX4IP
ASIC5	TLR8	ADAMDEC1	RASSF5	C1QB
FGR	FAM13B	LOC483397	PTPRJ	SLC41A2
GPR160	TRIM17	LOC482885	POLE2	KIF15
MS4A1	CD101	MND1	FBXO6	CAMK1D
PARP8	C27H12orf35	TROAP	GALE	WRN
EXO1	ENPP3	RHNO1	CDC42SE2	CD48
CENPA	TRANK1	KCNN4	NCF4	CLEC12A
BLK	EMP3	POLE	RPS6KA1	NIPA1
ZNF252	KLRD1	FAM65B	CCR1	DENND1C
PKIB	PSMB10	TPMT	TLR9	REL
CHN2	IRF1	MACC1	SNX20	MST4
LCK	PFN1	KNTC1	SLC27A3	HLA-DRB1

FSCN1	ACP5	KIF15	POU2F1	RAD51
GRIP2	CGA	NCF1	IL16	LY6E
CKAP2L	FAM57A	RARRES2	TREML1	LAPTM5
ITGAX	DERL3	MCM2	CSK	RGS10
RND1	RPS6KA1	DSN1	TNFRSF14	CCND2
C20H19orf66	GZMA	LIMD2	RAD51	MCM6
GLDC	IL16	CHRM3	PCDHGA3	CCR5
CCNA2	IFGGC1	CD48	SLC51B	UNC119
LEF1	SLC16A6	MCM3	TIMD4	CDC42SE2
TLR10	UNC119	GIMAP7	IFIT3	CEP152
THEMIS2	LYSMD2	NAPSA	PAG1	TNFSF9
ARL5C	CYFIP2	CSK	PCDHGA1	CD69
FAM65B	RASGEF1A	WFDC2	RGS10	TRIM59
KPNA2	SHMT1	FEN1	BCL2L14	APBB1IP
ITGA4	COTL1	ZDHHC14	POU2F2	DLA-DMA
CHRNA9	LFNG	RMI2	VCAM1	PSMB10
FANCM	POLR2A	OLFM1	SMCO4	CDKN3
CHEK1	TRPV2	HMGB2	FZD2	CASP12
BLM	GALNT12	ACP5	HCLS1	NCAPG
CD48	RNF125	CD86	RARRES3	DENND2D
WRN	CD38	TRIM34	NCAPG2	STK17B
CASP12	ATP2A3	LOC490356	EARS2	VRK1
PLTP	ALKBH4	LTB	CDCA3	LOC488248
CCL3	IGJ	KIF11	PSD4	VAV1
SWSAP1	CCL17	LOC488248	VWA5A	BEX5
SYK	SRGN	C1QA	HTR3B	NUSAP1
IFI44L	SORL1	ZNF215	PSMB10	INCENP
RACGAP1	TMEM204	DLA-DRA	PDE3B	HCLS1
DNASE2	PYGO2	PTPRCAP	CCNA2	BIRC3
ARHGAP11A	CDC42SE2	IKZF3	CD101	
LOC100856122	RGS18	BTK	SLC41A2	
ANKS1B	GNPDA1	GSG2	EAF2	
CD22	HCK	FCRL1	POLE2	

BCL11A	LOC612422	PLK1	DHX58
OOEP	TRIM59	CYFIP2	TRAF5
TAF15	MARCKSL1	DPY19L1	NAPSA
BIN2	SPTY2D1	PFN1	NSMCE2
OMG	PAG1	CCR4	TEC
SLA	ARF6	MCM6	NCKAP1L
SPOCK2	DUSP5	LOC100686614	IFGGC1
CLU	PARVG	PMAIP1	SPC25
FABP7	RUNX2	ARHGDIB	DLA-64
LOC610699	ITGAX	MEOX1	MILR1
CD3D	IL17RA	BTK	NFKB2
MCM5	FMNL1	CCDC63	PREX1
SQLE	MIS18A	KPNA2	GRAP
NCKAP1L	IKZF1	C7H1orf112	DOK3
MS4A7	PARP8	CD101	PSPH
IL27RA	STK4	CHML	CD82
DLA-DQA1	RASSF2	CD3E	LY6E
PMAIP1	SASH3	AURKA	LCP1
LYSMD2	ZNF354A	ASIC5	FERMT3
CIITA	DGKA	CACNB4	ISG20L2
CASP8	DLA-64	ANKS1B	LYN
LRRC46	PARP12	OOEP	TMCO4
PLXNC1	FASLG	TRAIP	UBE2L6
FAM72A	ADD3	PTPN6	CAPZA1
RPS26	TBX20	CKAP2	ACTR3B
SASH3	NRG2	APOBEC3H	LOC476070
TRIM59	EPB41	FUT8	LOC481744
KCNA3	CD3D	TRIM22	TGIF2
CARD11	NUCB2	CSK	DNASE2
FEN1	SDF2L1	C4H5orf34	FES
NHLRC3	LCP1	SLC15A2	CCNF
GSG2	FMNL1	PREX1	CIITA
DSE	KCNMB4	SCAMP4	AP1S1

SNX10	TLE3	RND1	TNFSF9
AURKB	APOBEC3H	KPNA2	FABP2
FERMT3	DEF6	SPI1	GRB2
GPR114	DLA-DMA	MLLT11	SYTL1
GBP1	LYZ	INCENP	PBK
LY86	MLLT11	PFKP	B4GALT6
PYGO2	PTPRCAP	CENPL	PLD4
SUCNR1	CXCL13	RASGRP1	CXCR5
ACTR3B	VAMP1	CD33	GPRC5D
PTGS2	SPOCK2	KLHDC9	NCAPH
AK7	CASP8	GBP6	DEPDC1B
ARSB	BIRC3	VRK1	MLF1IP
KYNU	THEMIS	LOC100684769	PLK4
HCLS1	ANKDD1A	ACRBP	F12
COTL1	CORO1A	OMG	DTX3L
PTPRJ	PTPRC	DDB2	KPNA2
KCNA2	PLCG1	IRGM	UBFD1
MX2	IL18	CIITA	KIF11
PLD4	HCLS1	ARHGAP15	CD70
ACRBP	CIITA	PSMB8	PTPRC
LOC100684769	SLC41A2	BLM	HTR6
TNFRSF9	RAB37	RAP1GDS1	KIF4A
CXCR4	KIAA0513	CDCA2	PARP8
ISG20	NFATC2	TNFRSF14	LOC403529
ZNF292	FLT3	CEP72	RACGAP1
TP53I3	NCAPG	ITGAX	ABAT
RARRES3	CPLX3	PPP4R4	NFYA
ELL3	TNFSF9	FBN2	CENPI
WSCD2	PQLC3	LRR1	GPR18
CEP152	CAP1	ZMAT1	REL
OAS1	TSPAN5	UBFD1	NIPA1
NR0B1	CREBRF	ELAVL2	SNN
AP1S3	C20H19orf35	ARHGEF39	TNIP2

cOR52H7	TNFAIP8	CD40	BTK
CCNF	SORL1	TLR7	KIF23
MELK	EDEM1	OOEP	C11H9orf91
FUT8	ANXA3	LYN	DSE
EAF2	ANKRD50	CYBB	RRP1B
DLA-DOA	NMB	GINS1	TMPRSS7
USH1C	SLC7A6	LOC479820	GPSM3
GMIP	LRRC70	MASTL	RCC2
LRRC8D	CTRL	FAM49A	UNC119
OTUD7B	TTF2	SWSAP1	LPIN2
PRMT8	TPST2	UNG	PARVG
ZNF16	TIPARP	RRM2	LPCAT1
ORC6	CSNK1G1	DDX58	PARP11
RHEBL1	EBI3	NME1	SPI1
MLF1IP	AK8	SLA	KIF2A
COTL1	PSMB8	CCR6	PRR11
ITGB2	LOC481744	IL18	SPOCK2
LCP1	SRCIN1	LYZ	UBE2T
PTPN6	SLC26A8	SHMT2	BLNK
COL19A1	IL21	PIK3CD	KPNA2
LYN	IRAK4	OTUD7B	GNAO1
HMGB2	PNLDC1	NUGGC	DCLRE1C
STMN1	GNPDA1	STIL	SPOCK2
KIF15	GRK5	FAM221A	CENPF
MARCKSL1	GMIP	RAD18	APOBEC3H
RAPGEF6	PIK3CG	CKS2	CARD11
SEMA4D	CSK	CACNB4	RILPL2
PML	IRF8	TRIP13	ITGAL
DEPDC1B	FGR	RANGRF	GNPDA1
PREX1	ARHGAP15	C20H19orf66	ARHGEF39
MSMO1	UCP2	P2RY10	SLC15A2
SKAP1	WBSCR17	FBXL14	C7H1orf85
INCENP	PLBD2	MBOAT1	RIN3

LOC479820	REL	AUNIP	PRELID2
PRKCB	ROCK1	CD79B	PFKP
CYBA	GRAMD3	ZNF252	DNTT
CD74	LRIT1	LRP11	C6
PARP14	TNFRSF14	cOR6M4	DEFB123
RTN4RL1	LHX2	TSPAN1	NUF2
CCND2	DDIT4	STAP1	LOC100686614
MND1	DOCK11	CD19	MXD3
TMEM173	LOC477182	DERL3	C27H12orf35
UNG	CD48	NACC1	MBOAT1
GDPGP1	WFDC2	SKAP2	IRF8
PAX5	ETS1	CD72	CHEK2
GP9	CD97	KIF2C	GMCL1
KIAA0226L	PKLR	COTL1	RABEP2
ZDHHC14	RBM11	CEP55	ANAPC1
DNAH9	PRF1	TRABD	ERICH1
GINS2	TMEM231	GLRA1	MYO9B
SCG3	CEPT1	FAM60A	SURF6
CTSS	SEC61B	ARSB	CD40
SLC41A2	CTRL	CARD11	SLC46A3
LYN	DDX26B	LYN	SGOL2
SCG5	PTPRE	PSAT1	MFNG
PLBD2	C25H8orf58	LOC477558	SWAP70
TNFSF9	ARHGEF3	IL2RA	ARHGAP9
MASTL	TNFSF11	POLA2	KNSTRN
FIGNL1	TAF15	STK17B	TBC1D30
GPR34	GPSM3	ITGAL	NAGA
NOP58	FERMT3	CD5L	CDHR2
EVI2B	CCL3	PSMB10	STOML1
ARHGDIB	DTX1	GALK1	IFI6
BLNK	KLRK1	MIS18BP1	DLGAP5
RGS1	CYTH1	POLH	NIPA1
KIAA0513	RAP1B	PARP11	DGCR8

TMEM154	ARF6	DLA-DMA	BLM
PSD4	ZNF217	DOCK8	SSH1
TMEM140	ANTXR2	CD22	RIPK2
TFPI2	ERLIN1	LOC489372	SEMA7A
GRAP	SEMA4D	ALOX5AP	STAMBPL1
PSMB10	TMEM187	PARVG	IFI35
TLR3	CDKN3	APBB1IP	RASGRP2
MXD3	AKNA	LOC481744	EIF5A2
HPSE	ADCY7	LYSMD2	SLC6A6
ORC1	S100PBP	SAMSN1	LPCAT1
CYFIP2	PSME2	FAM187B	TRIM34
ENO2	PFKP	TMEM216	GINS4
PEX26	TNIP3	MGMT	SKA3
BPIFB1	ISG20L2	RPL34	FUT8
FAIM3	TFEC	PARP9	PPP1R12C
PSMB8	COL19A1	POLE2	PTPRCAP
TAP2	PEX26	UPB1	DAPK1
NUGGC	PDE4B	LOC609038	IL6
KIAA1467	GRAMD1B	CD40LG	PSMB8
CDCA2	MIER1	CD6	MASTL
HELLS	KLRD1	CKAP2L	SPIC
GALNT3	AR	TFAP2B	TP53I11
SKA3	CLEC4E	DNAH8	SGOL2
ABAT	TGFBR1	PCNA	G6PC3
PTPRCAP	CYP2D15	PLD4	CATSPERG
SLA	PARP9	PCDH8	EBI3
CD3G	SAG	STX11	TMEM173
HMGCR	C32H4orf32	PLBD2	DEPDC1
TAP2	RGS6	BPHL	CNTN5
GSAP	ABCB1	BLNK	GMIP
CD37	ARHGDIB	FAM26F	C31H21orf91
C7H1orf112	CLNK	CECR1	SYK
LPCAT1	DNAH3	CHN2	SHMT1

CKAP2	DTX3L	LY6E	SEC23B
UNC13D	CCDC117	CD2	MITD1
CMA1	TNFAIP3	PLXNC1	MIS18BP1
CTBS	DGCR8	LY6E	ARHGEF2
LPCAT1	IL12A	MAP4K1	CCNB3
AURKA	RRAS2	DYRK3	FGD3
KIF11	IFI16	SIGLEC10	OSGIN2
RAB20	DCP2	DLA-DOA	GFI1
PTPRC	CAMK1D	LOC484934	PTDSS1
USP18	RRAS2	SULT1D1	WRN
IFT122	LOC476825	EZH2	DLA88
FXYD2	PSD	TFEC	PAX5
POC1A	VAV1	KIF15	BANK1
KIAA0895L	IL27RA	TLR10	SETDB2
IKZF1	ACTR2	LOC491107	BANK1
LRMP	LOC479975	RPS26	ACOT12
GMCL1	GALNT10	IFI35	SH2D1A
CSF1R	TESC	CD79A	FUCA1
SGOL2	ATAD2	ELANE	LOC607937
TAGAP	SEC23B	CDHR2	PWP2
APOA1	TMEM154	TAPBP	CIAO1
RARRES2	DNAH2	DNASE2	ECT2
RCVRN	EFCAB3	BCL2A1	CD48
MYL4	ERN2	TUBA1B	BTLA
DYX1C1	IL13RA2	HEY1	WDR4
GUCY1B3	HDAC1	LOC491550	PXK
RHBDL2	ATP6V1E2	SPOCK2	TMEM206
C7H1orf85	PMEPA1	LOC485220	VASH2
COL1A2	STK10	TIMELESS	IL18
SAP30	MAP3K1	KIAA1598	TEX14
FKBP10	DAPP1	C9H9orf9	CST7
CD300C	CSK	CCDC109B	SLC2A6
UBFD1	LOC610177	ENPP3	RAB11FIP4

MCM2	FCER1A	TP53I3	GNPDA1
LSAMP	LOC484934	ZWILCH	RAB31
DAPP1	ZBTB7B	DSCR3	CEACAM16
LOC479668	PLSCR3	BCL11A	EMP3
DENND2D	PDCD1LG2	KCNA6	MAP4K2
SIGLEC10	ARHGEF6	CD3G	LOC608103
CENPK	RAB7L1	CEP89	NME1
TBC1D10C	CRLF3	TMEM154	TFPI2
AK8	B3GNT2	TRIM59	SPAG4
LCK	ACRBP	NKRF	LYPLA2
TRABD	RIN3	CRIP3	EXO1
SMAP2	PTTG1	KYNU	TTPAL
LOC483397	MOB3A	FASLG	TRIM14
CDHR2	ZNF323	CD37	SKA3
ABHD12	MBOAT1	LOC100686390	C1QTNF3
LTBP1	HM13	HAUS1	TGFB1
CXCL12	AK2	TBX4	DGCR8
LOC100685971	HEMK1	SMARCAD1	LRR1
PRKD2	FOXRED2	LOC610565	TAPBP
UMOD	TSGA10	CD74	ZUFSP
DEFB132	SH3KBP1	UNG	DOCK11
MYO7A	CDKN1B	FAM122B	BPHL
ARHGAP9	UBN1	RBM47	CD80
R3HDM4	GSAP	RFC1	FAM60A
TTYH2	LOC488258	TUBA1C	RAB11FIP4
TRIM34	NDC80	ERAP1	CD33
CD3E	MIER1	CEPT1	ARPC4
LRRK1	ABAT	CCNE2	SCAMP4
UBE2T	SCNN1G	LIG1	SIRT6
RAB42	LOC609039	TPX2	CDC25C
LOC485220	C1QC	DTX3L	PLEKHO1
CD27	SZT2	KBTBD7	TSTD1
NCAPD2	ARHGAP9	SKAP1	RBP5

IL16	FMNL3	TMEM144	TRAIP
VAV1	NCOA3	LOC488305	BATF
LOC100686309	FAM111A	KIAA1804	PIK3AP1
FAM117B	DOCK11	PAX5	MOB1A
TP53I11	LOC485601	SERPINA1	MLLT11
TIGD4	PDIA3	DLA-DQB1	PEX10
FMNL1	PARP8	IFI44L	PAPPA2
SERPINE1	LOC609235	IGSF9	RANGRF
NFKB2	TSKU	SPOCK2	FAM185A
GINS1	TF	EPSTI1	GALNTL5
SLX4IP	IL27RA	MILR1	ST8SIA4
CATSPERG	SMC4	CASP12	VRK1
SMAP2	RBP5	ACOT8	DSN1
TCF19	IQGAP2	LOC612422	FMNL3
LOC481744	GCA	LCP1	RAD18
FAM49A	SWSAP1	NASP	MOB1A
FAS	SNX10	ARHGAP30	AGER
GALNT7	LAIR1	SLA	PHF6
DBF4	CDADC1	LAPTM5	TAP1
PTDSS1	SLBP	ARMC9	DOK1
SGOL1	ITPR3	DNM1L	ENPP3
CTSH	STK17B	RPL22	GRIP2
CTSC	MGAT4A	FREM2	TRANK1
CTSZ	MAN2A1	NLE1	CHKA
ATP2A3	DENND1C	MTHFD2	ZNF292
ADRA1B	LOC479975	HEY1	RASGRP3
DOCK8	TAP1	TRAF5	SPSB2
SLC4A7	ABLIM1	HAUS8	LOC476669
CHN1	RECQL	POU2F2	GALNT3
STAP1	RBL2	SEMA7A	ATP6V0A2
MOV10	RSPO2	LEF1	SNX10
COL11A2	RIT1	URB1	RUFY3
NEK2	HSH2D	EVI2B	ZKSCAN2

ACOT8	CARD11	PPIL6	SGOL1
EZH2	SMCO4	MST4	C10H12orf56
KIF2C	CMTM6	PSME2	GBP1
CKS2	TOP2A	CD74	ADRBK2
SORL1	ERP27	MYL5	RND1
ETS1	PTPRE	CD3D	CDK4
CEPT1	SSH2	RAB31	CTSC
ZMPSTE24	PCSK1N	DBF4	CCNE1
C11H9orf91	TIPARP	PGLS	CENPL
ENPP2	RASA2	LOC610164	FANCM
CPA3	ABRACL	SASH3	PDCD1LG2
IL27RA	RNF32	FDXACB1	OSBPL7
CDC42SE2	PTPRO	RPS6KA1	MAP2K6
PIK3AP1	LOC491369	GRK4	SLC9A8
SEMA7A	FAM69A	PTPRC	TRAPPC10
POU2F1	AOAH	CAMK2D	DEF6
CRHBP	CAPZA1	M6PR	CTRL
PLCG2	CD9	FAM111A	PITPNM1
TOP2A	CFLAR	DNMT1	NEK2
CPXM1	MAP4K2	PHF6	SBSPON
SORL1	ACTR3B	KANSL2	HMGB2
DENND1C	ATP8A1	TIGD4	ARL4C
DDX21	TNFRSF14	THOC1	GPATCH3
RNF145	AURKB	ODC1	SFI1
NME1	SSBP4	CDT1	IFIT1
ERLIN1	HEMK1	PAPPA2	MS4A1
SKAP2	LAX1	COTL1	MATK
DNMT1	C8H14orf119	TEC	PIKFYVE
PRPSAP2	ACTR5	LOC480921	UBE2G2
ZNF133	MOB1A	UBE2T	CCDC50
TAP1	GDPGP1	PRR11	C9H17orf80
TRIM37	TBC1D2B	CCL5	CD3D
LOC403581	CREM	MXD3	RRM2

POU2F2	TMEM263	LOC100855609	EGLN2
DERL3	OSBPL7	DSCC1	RFX5
CDC45	GPR39	C2CD5	PRELID1
OLFM1	P2RX7	RSPH3	MTERFD1
ALKBH4	CLEC12A	R3HDM4	TPM3
RABEP2	GNMT	CBX5	ZNFX1
SGOL2	BUB1	AMD1	TOP2A
UCP2	LOC100688470	SERPINA1	ZDHHC18
TYROBP	TAPBP	CPZ	TMEM216
TTF2	CD70	PPIF	PHF16
SNAP23	UNC13D	TMEM130	SPATA2L
TNC	FLI1	FANCI	CHEK1
ST3GAL5	IL2RB	FAM185A	CERS6
CLEC4E	CDC42SE1	TCOF1	FAM189B
GFRA1	CDC25B	DLA-DQB1	HMHA1
RPS24	OAS1	FANCA	SNX2
KCNAB2	DDX26B	KBTBD8	S100PBP
MCM8	CCNB2	ZDHHC13	TPST2
KCNA6	LRMP	TF	CD1E
PARP8	CELA1	ANKLE1	MST4
CTLA4	LY9	CD80	HELLS
GRIN2B	SNX20	FIGNL1	SLC9A7
CHST2	ZBTB37	LOC488258	PPP6R1
NCAPG2	AGER	SLC38A1	TP53I11
XAF1	NUCB2	TAPBPL	STK4
LOC491550	ZUFSP	BIRC5	BLM
SAPCD2	IKZF3	MYCBP	FAM13B
CCDC172	PSIP1	PARP8	ICOSLG
CRYGA	APBB1IP	IL2RG	CDC45
EPSTI1	SPATA19	MYL4	ZAR1L
ESCO2	ST3GAL5	DCLRE1C	AP1S3
SLC6A12	LOC100684605	NUP37	MYCBP2
FGD2	ALG8	DNAH10	KRTCAP3

CNPY3	RAP2A	DPPA5	LOC482124
DNTT	WAS	MSH6	TRIM44
COL9A2	LRRC71	LPCAT1	TMEM150B
IFI35	CD44	SGOL2	RRAS2
MCM6	SPIC	RHPN2	CTSS
HERC5	FCHSD1	IRF4	FAM111A
CACNB4	TMPO	BCAT1	LACE1
GPSM3	DIDO1	CCDC150	EZR
CHRM3	LOC609540	SASH3	DGKA
IL2RG	TP73	KCNA2	CCNE2
LOC100688778	TMEM74B	PCDHA5	CCDC50
ELANE	EBP	TSHB	KAT2A
SUZ12	SLC5A3	KCNAB2	TMEM154
SLC2A6	ATM	NUP205	NFYA
LGALS9	ALOX15	TLR9	FAM190A
MYO5A	ELOVL5	ABHD12	SASH3
SDF2L1	NCKAP1L	SCN2A	HMGN1
FAM3B	TP53I11	MCM4	SACM1L
SASH3	DNMT3L	FBXL14	SASH3
LOC483842	ANXA9	ACSL5	PLK1
DLA88	MXD3	ATR	ERO1LB
RANGRF	LY6E	CCR5	LRRC25
EBP	NEIL3	RALGPS2	ACOT8
MYCBP2	ABHD15	THEMIS2	CFLAR
LOC609038	USP20	PTCHD2	ACTR5
BANK1	ALG10	MOS	SMIM14
HAUS1	ZPBP	HMHA1	SAP25
MFNG	ITPR3	MOV10	GPR34
FMNL3	TMPO	WDFY4	ANXA3
SRRM2	CNIH2	GPR39	CXCR6
LOC608103	HMGB2	LGALS9	SPC24
FCHSD2	ITGA4	RRP1B	MYO5A
RFWD2	NIPA1	NOP58	GNG13

ABCA3	EZR	CCNE1	CSNK1G1
PFKP	ARRB2	NUP160	SNX8
DSN1	RAPGEF6	TCF19	CD22
NIPA1	HECA	ZNF292	CPNE1
DGUOK	EP400	DIAPH3	WDFY4
NAPSA	CSNK1G1	ARHGEF6	LYZ
FCRL1	LRR1	C18H7orf72	PRKCD
LOC100684560	MAN1A1	TLR8	GUSB
CENPN	CARHSP1	POU2F1	STK10
MAP3K19	TRIM37	LOC476669	IRGM
RBM11	PABPC5	TUBB	DYRK3
NLE1	TP53INP1	KIF2C	POLH
PNLIPRP2	CLCF1	CFP	CCR4
BRINP1	MOB1A	TAGAP	CYTH4
POLE	IRF4	GMCL1	KCNN4
RAD18	PRIMA1	RAD1	SRRM2
HMHA1	TYROBP	WDR4	RFC2
PIWIL4	GLIPR1	TMEM173	KIAA0226
FOXM1	GLIPR2	FMNL1	KIFC1
FAM122B	B2M	WRN	ATAD2
TOPAZ1	CCDC88B	CTSH	GNRH1
DYRK3	FAM100B	PCNXL2	RRP7A
IRF5	GLI3	MITD1	SCPEP1
TRIM17	CBD139	LPXN	SIDT2
PSME2	KNSTRN	BLM	ZNF276
DLA-DMA	KBTBD8	DKC1	TEX15
LOC607281	ENO2	PTBP1	ATP10D
MAP4K1	PKIB	CRYGA	MYOF
BIRC5	KCNN4	RNFT2	TESC
DPY19L1	TMCC2	GALNT6	DTL
LOC610177	ZNF292	LETMD1	INTS2
IL33	RAD51	IDH3B	IRF5
IGFBP7	NIPA1	BRIP1	EZH2

MSMO1	EFCAB4B	CCDC77	FUT8
ADAM11	ABCG1	MSH6	FAM83D
AK2	IGSF3	DHX58	CDCA2
NRG2	CACNA1I	ITPKA	GINS1
APOE	MYNN	GBP1	NSF
RASSF5	UBASH3B	ZNF485	GINS2
UBA2	MYO1G	EIF2AK2	SLC35B2
ATR	GPR18	BRCA2	ARHGEF6
FUCA1	ZNF292	FKBP11	TRIP13
CENPL	MLF1IP	C21H11orf82	MYCBP2
TSEN54	FAM111B	RCC2	TNFRSF14
ANKLE1	EIF2AK3	MSH2	DENND4A
OAS2	SHD	ACTR3B	WRN
FAM126A	ESPL1	SLAMF1	ZNF292
ZBTB7B	KCNAB2	PRELID1	AR
GINS4	DSCR3	ATP6V1G3	LOC480921
DNER	RNF19B	DYX1C1	MTHFR
SLC9A8	RPS6KA1	GP9	ACTR2
CENPI	LLGL2	C27H12orf35	PARP8
DTX3L	FAM69A	SNN	TPMT
SPI1	SFMBT2	HMGN1	JRKL
DLA-DOB	PDE7A	LOC610699	IL33
ZNF488	LMNB1	WARS2	CHML
SCAMP4	AK8	FAIM3	LOC485601
CHST4	RELT	CCND2	CCR3
FAM185A	CHST7	IRAK3	PARP12
CXCR5	PSIP1	MTHFD1L	EIF2AK2
LRR1	CREB1	TSPAN15	ARID1A
GBP1	LBR	RECQL	BAX
CSK	IFIH1	SERPINB8	IRF1
CD74	LBH	TBC1D31	TFPI2
BANK1	HSD17B7	CDC42SE2	PSME2
LY6E	CCNF	TNFRSF14	PRKD3

FMNL1	TSEN54	PARP12	LOC607937
DHFR	CD200R1	LOC607281	PPIL6
TESC	FZD2	CYP7A1	APOE
SLC22A15	ADAMTS14	PWP2	FMNL1
FAM13B	GMFB	CCR3	GH1
ODC1	CD74	FAM175A	SSH1
RALGPS2	WRAP73	ZNF428	C8H14orf1
CHML	BAX	HPS6	CRYGS
CCDC109B	TXNDC11	RNASEH2C	CALCA
OTOP1	CREB1	NOXO1	IMMP2L
DNASE2	POLR3G	DDX21	CATSPERG
GMFB	P2RY10	SPSB2	FBXL14
CDA	RPE65	TNIP3	LOC479820
PDCD11	KRTCAP3	MIS18BP1	RRAS2
TRAF2	HAUS6	SLC23A1	CAPZA1
CLIC2	CDC20	PPIL1	PPM1G
ARPC4	CLIC1	RABEP2	KLHDC9
CXCR4	FABP7	TICAM2	ELMOD2
PARP12	ATAD5	LTA	ACRBP
MTHFD1L	CISH	RRP7A	MARCO
GUSB	NUF2	SPDL1	SLC24A5
RILPL2	EMR3	CSTF2	CKAP2L
RNFT2	CDCA7L	IFGGC1	P2RY10
TP53I11	EXO5	PRKX	AUNIP
LOC477182	VCPIP1	C1QC	DENND5B
LRRK1	HDAC1	TLR3	MAP4K1
GRK6	SBNO1	RAD1	SNAP23
AP3S1	SNN	PCDHGA3	RQCD1
ITGAL	HMHA1	RAPGEF6	P2RX5
FKBP11	TTYH2	CDCA8	IRF9
CPZ	PRKCZ	REN	INCENP
ETS1	ASPM	AHCY	ZNF513
C4H5orf34	DNAH9	GSTCD	SLX4IP

CAMK1D	GLTSCR1	E2F5	G3BP1
SEMA4C	KIAA0101	GPR180	APBB1IP
LOC488258	PTCHD2	ZDHHC13	PAFAH2
BLM	MMD	DHFR	MYO1G
LOC488248	TPCR63	SERPINA7	CTRL
GIMAP4	INTS2	SLC7A10	CXCR4
HAUS3	TAP2	NFXL1	C10H12orf66
NCAPH	GRIP1	SGOL2	NPRL2
CXCL16	TTC14	IMMP2L	GMCL1
BAI2	SLBP	KIAA0513	ELOVL5
FBXO5	CHSY1	TIMD4	SLC4A7
TUBA1B	STMN2	CDK4	SH3BP5
SLC15A2	ZNF276	WDR62	MYC
RASGEF1B	SHCBP1	SNRPA	GMFB
SAT1	LRMP	OSBPL7	DENND1C
USP1	RIT1	GALC	SLC25A19
P2RY10	GAK	PPP1R3E	U2AF1
DCLRE1C	PHLPP1	TRIM14	RASSF2
ZNF169	VWA5A	RYR2	WASF2
TMEM106A	TRIM11	SPATA17	CENPA
CDK4	IL33	RAD54L	ZNF16
PTPRC	GNA13	TYMS	HPS4
RGS6	GPR180	DOK3	FAM49A
TMEM86A	OPN1SW	WDHD1	CLU
RNF145	CYBB	IRF8	TTI2
RASGRP3	CERK	PSIP1	CD53
LOC607055	HSPH1	ZNF16	POLD1
ZDHHC24	LOC100688778	MYO5C	LY6E
CD40LG	USP48	CTLA4	ITGAX
REL	LOC488305	AGK	LOC607281
CEACAM23	ARG2	RPA3	MCM10
DLA-64	ELMO1	TNFSF9	ADORA2A
DUSP6	NUB1	CDC7	IKZF1

MCM10	STK39	SYTL1	CDA
C8H14orf1	DLGAP5	CEP164	ZDHHC13
PRKCD	TCEAL5	CLIC6	ADPRH
SPOCK3	FGFR1OP	CDK4	FAM26F
RPA3	VRK1	SLBP	UNG
HHAT	FLNA	LRBA	DNAH7
BTAF1	STK38	DENND1C	PUS7L
SEMA3D	UBE2J1	PES1	RNASEH2C
HAAO	ABCA3	RFXANK	ARHGAP24
RTKN2	CASC5	BRCA1	ARRDC5
RPL22	ANP32E	SLC41A2	DPPA5
MCCC1	SLC22A11	CBX5	TFEC
IL12A	SLC25A38	ZNF292	EBP
IFIT1	PCDHA5	TEX14	CEP170
KIF2A	LYSMD3	FAM69A	ALKBH2
NASP	PHF20L1	GUCY1B3	CCR7
PHF6	RPL19	WAS	MORC3
FBN2	MGA	CD27	ERC2
TUBB	C20H19orf66	HAMP	TMEM106A
ABCA4	SPAG5	NUDT1	ADRBK1
B4GALT6	TUBB	DTD2	BDH1
SLC23A1	FAR2	S100A9	IRAK3
GGT1	BTG2	ENG	BRIP1
KIFC1	SP4	AP1AR	IL2
HCK	FBXO33	LRRC8D	SDF2L1
ZAR1L	MSL2	LOC100687347	CXHXorf21
BAZ1A	TGFB1	DUSP5	CAPG
CCNE1	ARPC4	SRFBP1	CYTIP
MST4	NFYA	CBX5	DIABLO
GALNT12	TAF8	ENTPD3	URB1
IFIT3	TAP2	SLC6A12	WIPF1
TKTL1	ZDHHC17	WRN	SLBP
MAP4K2	TGIF1	TRA2B	BAZ1A

IL4I1	NCF1	GDPGP1	COL11A2
TLR9	C34H3orf70	TMPRSS11D	AP1B1
GEN1	SPATA13	CD300C	LOC403829
PARP11	SURF6	EIF1B	CNTRL
SH3BP5	C6H7orf43	TOP1	TNF
PIK3CD	FAM65C	FIGNL1	HIP1R
PRELID1	POLK	GNB3	CAMK2D
ARPC1B	VASH2	ALG10	MCM6
SPO11	OGG1	SRBD1	PLXNC1
DYRK3	CXCR6	LOC403581	PPP1R21
GOLM1	FAS	GABPB1	WDR91
ATRNL1	TPM3	METAP1	ARPC1B
ODF2L	FAM189B	ARMC5	MGAT2
FANCG	CENPL	ITFG2	CCL2
LLGL2	OPTN	LRRC8B	TNFAIP8
HIRA	PRDX4	SMAP1	SLC29A3
KIF18A	FYN	BAX	PHF10
IDI1	PDCD4	PMS2	LOC484934
CP	MFSD6	PCNA	TTC23L
MCM3	RGS17	DYRK3	PDIA4
TNF	GDPD3	DLA-12	HEMGN
CXHXorf21	HLTF	RRP1	CEP55
HSD17B7	SEC61B	SMCO4	PCED1B
LCP2	ELOVL5	NSF	KANSL2
GRB2	DPYSL5	CHAF1B	ITPR3
CXCL10	INCENP	RBP3	FBXO5
CECR1	CHD1	C1H19orf40	DENND2D
CCR1	PITPNM1	ADSS	CCDC102A
TRAF4	PRR11	E2F1	TTC9C
LOC100855609	IPO11	CYP2D15	GGA2
SMCO4	ACTR2	PPA2	SKAP1
CASP4	OSTF1	C13H8orf76	TCF19
DUSP5	CHMP1A	PLEKHA5	C9H9orf9

CUBN	TMEM176B	SMC4	GNAI2
SLC38A1	LOC476445	HCLS1	WDR37
IKZF3	ABLIM1	KDM5D	TTC7A
MMD	LOC100856565	TEX30	MRTO4
STK39	PNMA1	RBM15B	LTA
C1R	SLC2A6	EXOSC3	USP48
PABPN1	CDCA3	KIF18A	XKR9
CDKN2AIPNL	GTF2H3	AMICA1	P2RX7
PLEKHA2	GABRG3	DGCR8	GALNT7
DCP2	NUSAP1	CSTF3	GATSL3
NUP93	TUBA1B	NDC1	NUP93
RABGAP1L	ACTR3B	MGME1	GBP1
FGFR1OP	ADAM11	EZR	CFL1
CCR6	RILPL2	ZDHHC24	PLA1A
LOC100856041	CBLC	PDIA4	DENND5B
PFKL	C5H11orf57	PRKD3	TAP2
APBB1IP	ZNF783	LRMP	CCL8
CD79A	GPATCH3	RAN	PCDH8
RRM2	SUCO	BID	TOMM34
SLC30A1	SPOCK2	NAGLU	SPATA19
SPAG4	BPTF	CLDN9	ANP32E
ARHGEF2	XAF1	CTRL	FMNL1
CDK20	FAM118A	STK35	MFSD6
POLE2	BRAT1	PDSS1	PYCRL
CD4	RBM14	ARHGAP9	CREB1
SETDB2	TNFRSF9	NUP43	TAGAP
BCL2L14	TBKBP1	CDA	C4H5orf34
F5	NLRC3	VAV1	CCT8
PRKCZ	PCNA	MMP12	LOC611606
TUBA1C	SMCR8	CD160	CAPN10
WDFY4	CDK1	CDKN2AIP	ERCC6
LOC612422	ARHGEF6	VAV3	THOC1
MSH6	SBNO1	EBNA1BP2	MCM2

ARHGEF39	YWHAQ	KIAA0391	TCOF1
PRELID2	CAPN10	PRR22	ZSWIM3
LIPA	DRD2	WDR62	LOC479974
E2F8	BNIP3L	RPL22L1	CCDC6
CHTF18	ST3GAL4	QTRT1	POLA2
FAM71D	FAM187B	MRTO4	TRIM25
WDR76	SEMA4C	AK2	OTOR
ADPRH	KIF23	SAP30	CCL4
B2M	SLX4IP	AIF1	EIF1B
MBD5	LOC484323	CASP2	STXBP2
TMEM231	HERC3	RPA3	RFXANK
FGR	ESYT3	EBI3	PRPSAP2
C27H12orf35	MTF2	TLX1	SMC4
TRIP13	FEN1	GALC	SKAP2
TMEM216	AK7	RANBP1	SDC1
DRAM2	CHST11	PRIM2	LRRC36
PQLC3	GINS4	CIAO1	GNAI2
PLA2G4F	CCL22	NETO2	DAPK1
ALOX5AP	EBP	PCED1B	HSD17B4
EMP3	RNF213	HERC5	MYD88
RASGRP2	CCNA2	INTS7	SLC19A1
SLC36A4	PGS1	CTSS	FEN1
CDH2	SCAMP5	LRRC71	CPO
E2F1	RPA3	WDR89	TARBP1
BST2	AQR	ZNF75D	HEMK1
MMP12	AP1S3	CASP12	GBA
LIPA	CTSC	IGSF6	FCHSD2
SUPT7L	LPCAT1	NUB1	ADSS
CST7	STK35	TMEM107	MCM5
GALC	PRRC2B	ADORA2A	CNPY3
ARHGEF38	HP1BP3	NOC3L	MORC2
WDR4	NEK2	PPAT	CD300C
C34H3orf70	ITGAE	UHRF2	RNFT2

IRAK1BP1
PGLS
CLEC9A
COR2T18
NIPA1
SEMA5A
NUP37
PIEZO2
ATP6V0D2
DOCK8
MFAP1
ZDHHC20
HSPA4L
POLD4
HSPH1
HAUS3
SLC16A11
DGCR8
LCTL
LOC403529
GPR114
CDC6
SS18
FIP1L1
C1QB
RGS2
LOC100684605
OR10A4
DOCK3
VIL1
PHF23
CREBRF
FGR
KRT18
BTBD11
ANLN
CDKN1B
EZH2
CREB3L4
FNDC3A
NELL1
TRIM4
MYO5A
NOL9
LOC485601
MPHOSPH10
MYOF
ICOS
C9H17orf80
SYK
SPAG5
MYD88
DCTPP1
DDX47
LPCAT1
LOC490917
PSD4
POLR2A
BFSP2
CD83
TTLL1
SCML4
RILPL2
LOC474791
GALNT7
AEN
DEFB122
LDHB
MCM7
OAZ3
CFL1
LPIN2
LIPT1
TSEN2
F12
TMEM107
UNC13D
CEP72
ARMC5
L3MBTL3
HAUS8
FBXL14
TLR8
STAT5A
LGALS9
IL27RA
MIS18BP1
SLC14A1
ATR
ZWILCH
AHCY
GPN2
AKR1C3
ALDH16A1
MTMR4
SPEF1
AAR2
PPIF
GLA
LOC610565
TMEM176B
UCP2
GALNT10
TTC21A
ABCC1
DNAH5
A2M
SPHK2

GLCCI1	CASP4	HPS6
FLT3LG	GUCY1B3	LCMT2
NCR3	SMIM8	CD80
SLC38A1	MARS2	ASIC5
OAS3	MAP3K8	MGAT1
S100A2	SLCO2A1	ENTPD7
KIF15	DRAM2	SMARCAD1
AKAP13	TPM3	ALOX5AP
SAP130	CELA1	PPAN
LOC483397	GOLM1	TRIM59
C34H3orf70	FASTKD1	B2M
FBXO4	CTSO	E2F8
MADD	TDG	AIF1
RBM12	CASP10	GRIN2B
LOC477558	OSGIN2	SERTAD4
RPL27A	CHRM1	TST
CYBB	CXCR6	CENPK
CHD3	ECE2	DHCR24
CD83	TRAF4	DENND3
ADRBK1	CENPQ	TRMU
SHFM1	SPIC	HEMK1
ABCC10	TSTD1	ZCWPW1
REN	SLC25A32	CMPK2
IL4I1	NIN	ZC3HAV1
UBE2W	DGCR8	NOC4L
UTP6	POU3F4	NUSAP1
PKLR	CEP152	FAM49B
FAM49B	PPCDC	RPA2
CKAP2L	NIPAL2	CHRNA3
SKA3	C10H12orf66	HTT
LOC403581	GNGT2	CDPF1
HOGA1	SREBF2	ZNF252
PLCB2	ANXA3	UGGT1

CCDC11	RBM14	GIT2
ZNF614	UBLCP1	PLEKHG1
APAF1	EXOSC2	NDE1
IL25	G6PC3	CEP250
XYLT2	ADPRM	DDX20
CSPG4	EIF5A2	CDKN2AIPNL
ANTXR2	CAMK1D	AJAP1
MTHFS	TRIM50	CNR2
ARSE	STAR	PSIP1
MT4	EME1	TBC1D14
HAND1	ALPK1	MANBA
SPC24	LOC612071	RAB3GAP1
TTLL4	ITGAE	SLC1A2
ATP8A1	TGM7	DENND4A
OTOP1	MEN1	FTSJD2
MICU1	SLC25A24	CSRNP2
PLK1	TNFAIP8	SCAF4
PPP1R16B	FAM154B	ZCCHC12
ZMYND10	TLR7	ARID4B
FICD	DSE	FLI1
CFLAR	CD200R1	RUFY3
ZMAT2	IFIT1	LRRC14
PSD4	FAM13B	BCHE
LRRC59	LRRC59	NUP153
PHF6	PTPRC	BPNT1
PLXNC1	CERKL	SLA
PPRC1	WDR76	CREB1
A4GNT	B4GALT6	RNF145
NRAS	SRSF9	HHEX
FAM71B	UBE2J1	CTSA
TP53I11	NPPA	CSTF1
PLEKHA1	C8H14orf119	FDXR
NRSN1	FAM129C	SFT2D2

TMEM140	DQX1	PLEK
AHSP	DDX47	MFAP3
FAM64A	ODF2L	ARSB
ZNF800	NPM1	KIAA1370
ADSS	KLHL18	PIEZO1
E2F1	SLC25A14	LOC475708
SMEK1	PPM1E	POLE
PI4K2B	ITPR1	KIF5C
PIK3IP1	OSBPL5	MYL4
HAVCR1	GEN1	SH2B3
EDEM3	AKAP3	SLBP
ERICH1	PRKCD	GSKIP
ZC3HAV1	MATK	MTO1
MTERF	GCN1L1	BCL10
GRB2	ZNF323	ITPR1
TAGLN3	SWAP70	SETDB1
CREBRF	CLP1	C13H8orf76
LOC613008	FXYD2	CKAP2
FRYL	NUSAP1	TXNDC16
TRAIP	RASGRP2	TFEB
SUZ12	LOC102157036	DUSP5
MCM5	RASGRP3	NFKBID
ZCCHC6	SMAGP	PDIA5
FBXL14	LOC482691	RNF145
AP3M1	SHMT1	LOC100688778
NUAK2	UBA2	STAT2
C7H1orf85	SLC19A1	ZFC3H1
SEZ6L2	ERAP2	FAM175A
ABHD13	DLA-64	PAXBP1
S100A12	SNRNP40	AMD1
CCDC85B	TCEA1	IL2RA
KIAA1370	GMCL1	SLC7A6
TRA2B	FGD3	LACC1

MED15	SNW1	HLTF
VASP	ST8SIA4	CHST2
SUCO	PARP8	MEN1
PDE7A	APEX1	SENP8
PEX6	APRT	SNAP23
FGFR1OP2	STK10	G6PC3
ZNF711	EIF4E	ZMYND10
PFN3	HMHA1	BRCA1
SND1	PSMB9	CNOT1
ANGEL1	CRLF3	PKMYT1
POLH	NCOA5	SLC9B1
SLC10A7	MCRS1	SCAMP4
USP35	LRRC40	LRRC8D
STK39	GMIP	MCRS1
RAB8A	GEMIN4	cOR52H7
SLPI	EHD3	TROAP
DNAJB9	RABGAP1L	TRIM37
LOC481791	TIMM8A	MOB3A
SPC25	DHX36	CSNK1G1
LOC100855903	DCP2	C1QB
ALAS2	SLC25A16	LOC100688904
ZFP30	EARS2	FOXRED1
CCL4	RANBP1	DENND4A
KIAA1524	RIPK2	UBR7
ARID1A	KBTBD6	TIMM8A
IGFLR1	ILF2	PRKD3
TXLNA	PAXIP1	SRM
HUWE1	LSM7	MND1
LOC100855609	IKZF1	RARRES1
LOC484976	NOP56	GRIN2B
GSTO1	PAICS	BACH1
S100A9	CAPRIN2	CDC20
RAD18	GMNN	NR2F6

PHKG2	PRPF4	VANGL1
MSN	LACE1	MCM3
PIM2	SLX4IP	TMEM260
CNN2	DDX39A	IL18BP
SLC1A6	RPUSD4	C8H14orf1
SLC35C1	ATAD2	RRM2B
INPP5B	ARL6IP1	RPS19BP1
G6PC3	LOC483987	FUT1
TNF	SPTLC2	PIM2
KIF4A	FSIP2	SLC15A4
ADORA3	FGFR1OP2	POP5
PLD3	PLCB2	CAMK1D
PRPSAP2	FAM122B	CAMKMT
PSKH1	RAB27A	KNTC1
LOC607281	AP1S1	EVL
MGAT1	USP1	ZNF133
TRIM26	GK	ARPC2
SRPK1	LOC479974	RRP1
MIER1	DDX18	MTR
LRRC59	UBA2	FOXM1
GUCA2A	PRPSAP2	CAPS
FAM58A	THEM4	WNK4
LIM2	CPSF3	PCDHGA12
IFNG	NR1H3	SPO11
OVCH2	KLHL42	GPALPP1
RNF213	RBMX	RASAL1
KBTBD7	GNRH1	KCNAB2
GAPT	TBC1D10C	ALG10
PRKX	TGIF2	TRADD
POLR3G	TGS1	TRAFD1
UBE2F	ALKBH2	ATP6V1E2
GIT2	PTBP1	NAGPA
ORAI2	PPP1R12C	ALPK1

ESCO1	SFI1	RANBP1
SGTB	CAPZA1	HSD17B7
JAK1	CNP	PDCD10
CERS6	RBM15B	TSGA10
TCOF1	CNOT6L	TSPAN14
CPXM1	LOC485693	ETNK1
PTPN6	NCOA3	SRSF5
ATXN7	NOL6	USP49
TMCO4	PA2G4	RELT
FAM208A	R3HCC1L	IDO1
SLC15A1	CLEC4E	CDC25B
CYTH4	SRCIN1	NOL9
RNGTT	RAB25	TUBB
LOC485570	DHRS13	C8H14orf119
CNR1	C1QB	LGMN
DBF4	MAP4K2	RECQL
MYSM1	OAS3	MAPRE1
KMT2A	EXOSC6	ZNF133
SLC37A3	PIK3AP1	RAB25
RHOG	LOC403529	GIMAP7
MS4A7	UBR7	DPAGT1
TRIM62	SFXN2	CYLD
NFYA	TRAT1	ZNF252
IL10RA	EBP	SHCBP1
SPINK2	U2AF1	LOC607328
NCF4	FAM65B	SLC23A2
HDDC3	MIF	CAPN10
WRN	RPP25	KCNA2
CDK9	MTBP	TBC1D10A
GNG3	OR16D05	SUCO
KIAA1967	ADORA2B	FAM65C
MCMBP	STOML1	APOF
S100A3	CENPP	RELL2

MRPS26	TRMT10C	ZNF839
FUBP1	FAM103A1	PARP1
PML	SFXN1	YARS
GPR12	DDX52	COPG2
SUPT16H	TAP2	PI4KA
PRM2	FMNL1	BRCA2
LOC482235	RAD51B	RAB20
TRAM1	ARRB2	NKRF
STAMBPL1	PAN3	ELF1
FAM49A	MAT2B	HEMGN
MED23	SAPCD2	MANF
DCUN1D3	ASUN	GEN1
MXD4	NFE2L3	METTL2A
ARHGEF39	SF3B4	NUPL1
STMN1	SNX30	NCOA3
BCAT1	CEP78	NOD1
RFWD2	CNOT3	RAB8A
NLK	NDC1	ABHD1
LRRC41	TRIM28	WDR83
LOC611067	PRPS2	CBLC
GPA33	CLEC9A	IFIT2
NEURL	BCL10	SACM1L
COMMD10	MSR1	DPF2
ZNF513	MYO9B	STRADA
WIPF2	PYCRL	OOEP
KMT2E	POLR3G	RSAD2
CTSS	DDX39B	RASGRP2
ADAM23	TM7SF3	KIF15
STARD4	PDF	CSNK1G2
NCK2	EIF4A1	C7H1orf112
LOC612867	SRSF1	TUBA1B
PPP1R12A	ARHGAP24	BBX
HSD17B7	LRRC16A	IL2RG

RAB4B	UHRF1	UNC93B1
STXBP2	TFRC	RHBDL2
ZCCHC6	HDAC1	SLC22A15
OR13F1	CXCL10	PCNA
DCTN5	FGR	LMBR1L
CDC42SE1	WDR43	PRPH
P2RX5	PAK1IP1	NAT9
OR16G03	FOXM1	SLC39A10
LRRC8D	EMG1	EMB
ESCO2	NIPA1	KIAA1731
PLA2G4F	MARCKSL1	MEN1
KIAA1109	C9H17orf53	MOV10
TNIP2	CEACAM30	DDX39A
TBL2	ADPGK	C20H3orf62
TTC23L	RFC3	TANK
ARHGEF2	CDKN2AIPNL	PAPD7
GTF2H2	SLC35F2	TXLNA
UBA52	ERAP1	CD274
SCN2B	RAB8A	SLC37A1
MADD	DLA88	PML
CSRNP2	ZDBF2	ZDHHC21
PRDM4	TTC23L	GFM2
FOXC2	HMGA1	RFC3
KLHL11	ST3GAL5	RNF26
XPO6	GTF3C4	FAM122B
G3BP1	PML	ST3GAL5
ELMO2	NSUN5	EFR3A
KCNA5	APOF	NUP205
TEX36	SNX2	PRPF4
HELZ	SETDB2	GGT1
IL21R	SLC35A2	E2F1
CDKN2C	WDR75	ACTR2
ACTG1	METAP1	NCAPD2

VCPIP1	ABRACL	RAD1
IGDCC4	KIF24	ASTE1
IQGAP1	TXK	CLK2
PKMYT1	NPM2	FAM111B
LOC100856638	HMGB3	HAUS6
AKAP10	DERA	NIPAL2
LPCAT1	FANCL	OOEP
DGKZ	CDKN2AIP	MYL5
KLF12	CDKL2	HSD17B7
PREX1	IL27RA	WAS
NUFIP2	CYP51A1	ACAT2
ADAMTS10	GSKIP	RELB
SH2D3C	ASPHD2	BRPF1
TBXA2R	TCERG1	RNASEL
PGLS	PSIP1	R3HCC1L
CAB39L	DLA-DQA1	NR1H3
RNF24	NUP85	MYCL
PBXIP1	H2AFZ	TTLL12
TTC31	ZUFSP	RASSF3
CYBA	GMFB	OLFM4
C6	MCRS1	PNPLA6
ARID4B	UNC13D	MSN
GPR34	TSEN54	CDH11
SAG	APLF	RANBP1
KDM2B	CD1A6	SUCO
NFKB1	SDF2L1	AGPAT1
RANBP9	REL	LOC608103
GDPD5	TK1	MFSD5
ZNF449	METTL14	ANKRD12
NCAPH	BAZ1A	PTPN1
SGOL1	PAAF1	DCK
HAUS1	KIAA1524	WDR16
PIWIL4	TEX15	UBA2

PARN	ABAT	FBXW9
ZNF667	POLR3G	DCP2
ADNP	HS3ST3A1	SLCO5A1
UPK3B	FGD2	FZD5
RFTN1	MYC	SRFBP1
BAG4	CCT6B	ELF1
OPTN	SLC25A15	SPAG11B
LOC403530	SEC23B	GALNS
RS1	DIP2A	NANS
TRIM33	SLC5A3	GOLM1
SEMA4A	GRAP	ZZZ3
RAPGEF6	TOMM5	CNTNAP1
FRYL	RPS3	APLF
GMCL1	GNS	ASPHD2
TTC7A	CDK5RAP2	IDH3B
THEMIS2	APAF1	ODC1
UBE2C	HNRNPAB	ARG1
PAK1	RPTN	ZNF485
PPP4R2	DENND2D	ITGA4
GLYATL3	MYB	SMAP2
MPEG1	TFAM	KCNK5
OTUD7B	CD274	EIF2AK1
SP3	LY9	TMEM222
ELF2	CTRL	LOC609039
ELF1	SNX12	SLA
LOC100855593	ZNF513	COG4
ALG1	RFC2	FDXACB1
PRND	TCEAL5	MCOLN1
TOP2A	THUMPD2	GSTA4
PHF6	FAM58A	ATXN3
GLRX	NXNL1	WDR46
GNG2	ITGA4	SLC35F5
TET1	WDR34	GRK5

RICTOR	PTPN1	BCAT1
TBC1D24	SPO11	SMAP2
FASTKD5	CDCA7L	KMT2A
ZNF366	OSM	AGPAT3
PTBP1	CTSZ	MAP3K1
DBP	HAUS3	SMAGP
MCM6	PCTP	SLC25A24
FANCG	NUPL1	SYCP2
RSPRY1	CEP152	RAD1
DAZAP2	IRF5	NOC3L
LIG4	PDCD11	NDEL1
GNB1	WRAP73	UFSP2
XIAP	BUB3	MAT2B
CCDC85B	TRIM37	B3GNT2
ATP8B2	HCK	RGS18
ATP6V0E1	LOC491839	COLEC10
FAM117A	FAM49B	LRRC40
ACTN4	PRKD2	TAP2
RORA	ARF3	FKBP15
EFTUD1	CHTF18	KIF15
DIP2A	F5	CNOT11
ABHD15	UTP14A	C21H11orf82
RBM23	ACTR2	GPR180
RACGAP1	INTS2	KDM2A
HAPLN2	TFAM	CXCR4
CD74	POP5	OASL
ALKBH2	INTS10	ST3GAL4
FAM117B	CCT8	BPIFB1
NPHS1	NOP56	FCGR2B
GNPNAT1	MOB1A	BATF3
KIF5C	SPTLC2	EBAG9
TP53	LAIR1	TCEA1
PLSCR4	NUP153	CFLAR

ACTRT3	SLC22A15	EXOSC6
SPATA2L	SLAIN1	ARID1A
LTA4H	TBP	RTN4RL1
GMPR2	MED23	DIDO1
USH1C	RQCD1	UHRF2
EVL	XRCC2	ALKBH6
G3BP2	TRIM37	PRKX
RLIM	LOC610614	USF1
GPR152	CAPS	DNMT1
FMR1	ACAT2	ZNF227
MAP4K1	UTP15	LOC482691
TBC1D10A	LOC100856713	CDC6
RCSD1	EXOSC7	SEL1L2
ZNF770	DDX23	CDC42SE1
LOC100688904	CNPY3	HNRNPAB
SNRNP48	CDK20	COASY
KIF2C	POLR1B	RPP25
MGAT2	CAECAM1	SIN3A
ZFP30	SMC1A	CDT1
LOC487366	SGK196	TMEM30C
MAD2L1BP	S1PR1	ETS1
EFR3A	NMBR	ACSL5
CDC7	GLA	ITPR1
IL1RAP	RPA2	RHEBL1
ECT2	P2RX5	MED6
NCAPD2	MS4A1	DNAJB7
HAMP	GPR173	FYB
ENTPD7	PINX1	ITSN2
CRIP1	NAT10	SHARPIN
KLHL8	STK4	SMC1A
CYTH2	CLEC12A	LOC482885
SOCS4	DNM1L	ARID4B
FAM107B	FANCE	GABPB1

MFSD1
MCM10
LSP1
RFC1
SLC7A10
LOC479974
CEP152
ARPC4
PIK3R1
ARHGAP12
YARS
MPEG1
UBE2W
IFT140

ZNF526
PAICS
ARPC4
FRG1
ARHGEF6
ELOVL5
SMAP2
RBM12
POLD3
SEC61B
LOC612106

LDB1
STK35
TLR7
SMAP1
OSTF1
PSME1
XPO6
ZNF614
SLCO1A2
KDM2B
GON4L
PPP2R3C
UBA2
LOC474791
LOC483987
C30H15orf57
ZNF75D
CHPF2
PCDHGB7
HMGN2
SCG5
OVGP1
DDX5
PAK1IP1
RMI2
ZFHX2
DIP2B
LRRC24
PISD
C9H17orf80
UBR4
BTAF1
ZBTB49

TLK1
SLC31A1
AK7
ZNF215
CNP
LGALS8
RBM27
GDPGP1
PHKA2
ABRACL
HK1
PPP1R9B
TAF6L
DSCR3
HERC6
ILVBL
FBXO18
KCNA6
CKS2
MYO19
PHKG2
ARHGEF6
ADAMTS10
STIM2
RAB27A
RNF38
SLC23A1
UTS2B
KCNA3
BUB3
VWA9
LOC100856122
SMARCAL1

CTH
TRAF3
ORC6
KIF18A
MSL2
FXN
CIAO1
CLP1
TSEN15
DAP3
PPRC1
TM2D2
CLCN7
PSIP1
MYEF2
SGOL2
MTRF1L
CD79A
BYSL
ENTPD1
C27H12orf57
SLC7A10
CEP152
HEXB
ATP2A3
AP1M1
USP1
SESN3
MVD
ZNF335
GRIN2C
ZDHHC24
RYR2

INTS7
GNPNAT1
CDKN1B
MBD4
ATM
TRMT112
SNN
KBTBD6
SLC25A32
CHD3
CAFA-T2R67
BFSP2
ZBTB17
ATP6V0E1
ITPR3
ERAP2
VPS18
WDR43
CCL28
FAM134C
C12H6orf70
LOC100855485
SLC25A16
FANCG
PGAM1
SLC25A1
SNRNP40
SPAG5
SFXN1
PYGO2

Supplemental Table S2: 146 genes identified from a 290 gene set in common in the 4 MDR canine tumors by a Cytoscape analysis to be highly interconnected.

Clusters	Nodes in Cluster	Node %	Node List
0	38	0.2603	APBB1IP, CARD11, CD2, CD33, CD3D, CD48, CD53, CD74, CD80, CD86, CSK, CTSS, CYBA, CYBB, DAPP1, FGR, HCLS1, HLA-DRB1, IDO1, ITGAL, KIF15, KIF4A, LAIR1, MAP4K1, NCF4, PIK3CD, PLCB2, PRKCB, PRKD2, PTPN6 ,PTPRC ,SASH3 ,SELL ,SEMA4D ,SH2D1A ,SLA ,TRIM22 ,VAV1
1	30	0.2055	AURKB, BUB1, CCNA2, CCNB2, CCNF, CDC20, CDK1, CENPL, CEP152, DLGAP5, DTX3L, ECT2, ESPL1, INCENP, KIF23, MIS18A, NCAPD2, NCAPG, NCAPH, NDC80, NEK2, NUF2, PLK1, PML, RACGAP1, SPC24, SPC25, STMN1, TUBB, VRK1
2	17	0.1164	BCL2A1, BIRC3, CASP8, CD83, CORO1A, FASLG, FERMT3, FMNL1, IFIH1, ITGA4, ITGAX, ITGB2, LTB, PARVG, PMAIP1, TNFRSF14, TNFSF9
3	10	0.0685	CCND2, E2F1, EZH2, FEN1, NUSAP1, RAD51, SWSAP1, TRIM37, TUBA1B, WRN
4	10	0.0685	CCL19, CCL20, CCL5, CCR5, CXCL10, GPR18, GPR65, GPSM3, ISG20, P2RY10
5	10	0.0685	ARHGAP15, ARHGAP30, ARHGAP9, ARHGDIB, FAM13B, FGD3, GMIP, PREX1, RHOH, TAGAP
6	8	0.0548	PSMB10,P SMB8, PSME2, PTTG1, REL, TAP2, TRAIP, UBE2C
7	5	0.0342	ACTR3B, ARPC4, CYFIP2, NCKAP1L, TF
8	4	0.0274	GINS4, MCM10, MCM5, MCM6
9	4	0.0274	BATF, CIITA, IKZF1, IKZF3
10	4	0.0274	KCNA2, KCNA3, KCNA6, KCNAB2
11	2	0.0137	FYB, SKAP1
12	2	0.0137	EBI3, IL27RA
13	2	0.0137	C1QB, C1QC
	146		

Supplemental Table S3: Gene enrichment

Network Pathway enrichment, FDR < 0.001

Gene Set	Proteins in geneset	Proteins from network	P-Value	Nodes
Signaling by Rho GTPases	338	30	5.23E-13	CYFIP2, CDC20, PREX1, RACGAP1, NCKAP1L, PLK1, ARHGAP9, NUF2, RHOH, GMIP, VAV1, INCENP, FGD3, CENPL, SPC24, SPC25, FAM13B, NCF4, ARHGDIB, CYBB, CYBA ,ARHGAP15, ECT2, ARPC4, NDC80, ARHGAP30, TAGAP, AURKB, BUB1, FMNL1
Mitotic Prometaphase	175	18	3.34E-09	CDC20, NCAPG, NCAPH, PLK1, NUF2, TUBB, INCENP, CENPL, NCAPD2, SPC24, SPC25, NEK2, CCNB2, NDC80, CDK1, AURKB, BUB1, CEP152
Aurora B signaling	40	10	3.76E-09	STMN1, NCAPG, NCAPH, RACGAP1, KIF23, INCENP, NCAPD2, NDC80, AURKB, BUB1
Mitotic Metaphase and Anaphase	171	17	1.51E-08	CDC20, PSME2, PLK1, NUF2, INCENP, PSMB8, CENPL, SPC24, SPC25, PTTG1, VRK1, UBE2C, NDC80, ESPL1, PSMB10, AURKB, BUB1
Cell Cycle Checkpoints	246	20	2.15E-08	CDC20, PSME2, PLK1, NUF2, INCENP, WRN, PSMB8, CENPL, SPC24, SPC25, CCNB2, UBE2C, NDC80, CDK1, MCM10, MCM5, MCM6, PSMB10, AURKB, BUB1
TCR signaling in naïve CD4+ T cells	67	11	4.41E-08	PRKCB, HLA-DRB1, CD3D, MAP4K1, VAV1, CD86, CD80, CARD11, PTPRC, CSK, PTPN6
Neutrophil degranulation	418	24	4.92E-07	TBC1D10C, UNC13D, BIN2, NCKAP1L, LYZ, ARHGAP9, LPCAT1, CTSS, CD33, CD53, TUBB, LAIR1, COTL1, CYBB, CYBA, PTPRC, SELL, ITGB2, LRMP, ITGAL, ITGAX, KCNAB2, FGR, PTPN6
TCR signaling in naïve CD8+ T cells	54	9	6.72E-07	PRKCB, CD3D, VAV1, CD86, CD80, CARD11, PTPRC, CSK, PTPN6
PLK1 signaling events	44	8	1.50E-06	CDC20, PLK1, INCENP, SPC24, ECT2, NDC80, CDK1, BUB1
Interleukin-10 signaling	47	8	2.43E-06	CCR5, IL18, CCL19, CCL20, CD86, CD80, CXCL10, CCL5
Cell cycle	124	12	2.77E-06	CDC20, PLK1, CCND2, PTTG1, CCNB2, CDK1, CCNA2, ESPL1, MCM5, MCM6, E2F1, BUB1
Regulation of mitotic cell cycle	83	10	2.89E-06	CDC20, PSME2, PLK1, PSMB8, PTTG1, NEK2, UBE2C, CDK1, PSMB10, AURKB
Rheumatoid arthritis	90	10	5.84E-06	HLA-DRB1, IL18, CCL20, CD86, CD80, ITGB2, ITGAL, ACP5, LTB, CCL5
Interferon gamma signaling	73	9	7.60E-06	TRIM22, HLA-DRB1, CIITA, OASL, OAS1, GBP1, PML, PTPN6, TRIM34
Costimulation by the CD28 family	65	8	2.48E-05	HLA-DRB1, CD3D, VAV1, CD86, CD80, TNFRSF14, CSK, PTPN6

Biological Pathway (GO), FDR < 0.001

Gene Set	Proteins in geneset	Proteins from network	P-Value	Nodes
cell division	314	24	2.59E-09	CCNF, CDC20 ,CDCA3, NCAPG, SKA3, NCAPH, TUBA1B, NUF2, TUBB, MIS18A, NCAPD2, SPC24, SPC25, CCND2, PTTG1, NEK2, VRK1, CCNB2, UBE2C, NDC80, CDK1, CCNA2, BUB1, SPAG5
defense response to virus	164	16	5.29E-08	TRIM22, UNC13D, APOBEC3H, IFIH1, TLR8, PMAIP1, ISG20, CXCL10, PTPRC, OASL, DTX3L, OAS1, GBP1, MX2, PML, TRIM34

positive regulation of T cell migration	11	6	5.92E-08	AIF1, CCL20, CXCL10, DOCK8, ITGA4, CCL5
chromosome segregation	57	10	9.96E-08	ESCO2, SKA3, TOP2A, NUF2, INCENP, MIS18A,SPC25, NEK2, NDC80, SPAG5
mitotic cytokinesis	36	8	3.37E-07	STMN1, RACGAP1, PLK1, UNC119, KIF23, NUSAP1, ECT2, KIF4A
neutrophil degranulation	420	24	5.48E-07	TBC1D10C, UNC13D, BIN2, NCKAP1L, LYZ, ARHGAP9, LPCAT1, CTSS, CD33, CD53, TUBB, LAIR1, COTL1, CYBB, CYBA, PTPRC, SELL, ITGB2, LRMP, ITGAL, ITGAX, KCNAB2, FGR, PTPN6
T cell costimulation	74	10	1.05E-06	HLA-DRB1, CD3D, VAV1, CCL19, CD86, CD80, CARD11, TNFRSF14, CSK, PTPN6
immune response	349	21	1.31E-06	IL27RA, TRIM22, TNFSF9, TNFSF8, CTSS, CCR5, MAP4K2, IL18, VAV1, CCL19, CCL20, C1QC, CD86, GPR65, NCF4, TNFRSF14, CXCL10, CIITA, FASLG, LTB, SEMA4D
mitotic sister chromatid segregation	19	6	1.40E-06	PLK1, NUSAP1, NEK2, NDC80, ESPL1, SPAG5
positive regulation of GTPase activity	294	19	1.54E-06	PREX1, RACGAP1, NCKAP1L, EZH2, ARHGAP9, GMIP, VAV1, CCL19, CCL20, FAM13B, ARHGDIB, ARHGAP15, ECT2, ARHGAP30, DOCK8, TAGAP, CCL5, SEMA4D, RGS10
regulation of small GTPase mediated signal transduction	140	13	1.66E-06	PREX1, RACGAP1, ARHGAP9, RHOH, GMIP, VAV1, FGD3, FAM13B, ARHGDIB, ARHGAP15, ECT2, ARHGAP30, TAGAP
T cell differentiation	31	7	1.67E-06	PREX1, GPR18, CD3D, RHOH, VAV1, PTPRC, PIK3CD
response to virus	103	11	2.87E-06	STMN1, TRIM22,I FIH1, TLR8, CCL19, ISG20, OASL, FGR, OAS1, CCL5, MX2
innate immune response	408	22	4.21E-06	APOBEC3H, SH2D1A, MARCO, REL, IFIH1, MAP4K2, TLR8, C1QB, C1QC, SLA, CYBB, CYBA, CORO1A, HMGB2, PIK3CD, CASP4, DTX3L, FGR, TRIM59, CSK, MX2, PML
regulation of attachment of spindle microtubules to kinetochore	6	4	4.70E-06	RACGAP1, NEK2, ECT2, SPAG5
positive regulation of T cell proliferation	53	8	5.76E-06	NCKAP1L, AIF1 ,CCL19, CARD11, CORO1A, PTPRC, SASH3, CCL5

Cell Component (GO), FDR < 0.001

Gene Set	Proteins in geneset	Proteins from network	P-Value	Nodes
Ndc80 complex	4	4	9.50E-07	NUF2, SPC24, SPC25, NDC80
midbody	119	12	1.80E-06	RACGAP1, RAB11FIP4, PLK1, KIF23, INCENP, NEK2, ECT2, KIF4A, CDK1, AURKB, SPAG5, SHCBP1
spindle microtubule	36	7	4.38E-06	SKA3 ,PLK1, NUSAP1, KIF4A, CDK1, AURKB, SPAG5
cytosol	4087	103	9.38E-06	CYFIP2, LRR1, CCNF, ENO2, IKZF3, CDC20, PREX1, STMN1, TBC1D10C, TRIM22, PRKCB, UNC13D, PSME2, PRKD2, SKAP1, IDO1, CDCA3, NCAPG, NCAPH, RACGAP1, NCKAP1L, SH2D1A, PLK1, REL, UNC119, DENND1C, ARHGAP9, KIF15, IFIH1, NUF2, DLGAP5, IL18, RHOH, KIF23, GMIP, VAV1, APBB1IP, INCENP, HCLS1, PLCB2, DCP2, AIF1, FGD3, PSMB8, MIS18A, CENPL, NCAPD2, SPC24, CDKN3, SPC25, FAM13B, NCF4, CCND2, PTTG1, ARHGDIB, NEK2, VRK1, CARD11, DAPP1, ARHGAP15, CORO1A, CCNB2, PMAIP1, ECT2, RARRES3, UBE2C, ARPC4, NDC80, ARHGAP30, KIF4A, CDK1, DOCK8, PIK3CD, OASL, CASP8, CASP4, TAGAP, ACP5, DTX3L, KCNAB2, CCNA2, FGR, HSH2D, OAS1, ESPL1, MCM5, LCP1, BIRC3, PSMB10, AURKB, CSK, GBP1, BUB1, MX2, CEP152, PML, FMNL1, RAD51, RGS10, TRIM37, PTPN6, PFKP, TRIM34

tertiary granule membrane	59	8	1.23E-05	CD33, CD53, LAIR1, CYBB, CYBA, ITGB2, ITGAX, KCNAB2
condensed chromosome kinetochore	81	9	1.69E-05	PHF6, NUF2, INCENP, SPC24, SPC25, NEK2, NDC80, BUB1, SPAG5
centralspindlin complex	3	3	2.34E-05	RACGAP1, KIF23, ECT2

Supplemental Table S4

cluster number	cluster color	gene count	protein name	protein identifier	protein description
1	Red	88	KCNA3	9615.ENSCAFP00000031052	Potassium voltage-gated channel, shaker-related subfamily, member 3
1	Red	88	CCL20	9615.ENSCAFP00000015443	C-C motif chemokine 20 precursor
1	Red	88	IL27RA	9615.ENSCAFP00000024279	Interleukin 27 receptor, alpha
1	Red	88	PTPRC	9615.ENSCAFP00000016636	Protein tyrosine phosphatase, receptor type, C
1	Red	88	CORO1A	9615.ENSCAFP00000025203	Coronin, actin binding protein, 1A
1	Red	88	SPOCK2	9615.ENSCAFP00000021263	Sparc/osteonectin, cwcv and kazal-like domains proteoglycan (testican) 2
1	Red	88	CSK	9615.ENSCAFP00000026494	C-src tyrosine kinase
1	Red	88	TRAF3IP3	9615.ENSCAFP00000017575	TRAF3 interacting protein 3
1	Red	88	MIS18A**	9615.ENSCAFP00000037287	MIS18 kinetochore protein homolog A (S. pombe)
1	Red	88	GMFB	9615.ENSCAFP00000037078	Glia maturation factor, beta
1	Red	88	SHCBP1	9615.ENSCAFP00000005520	SHC SH2-domain binding protein 1
1	Red	88	PARVG	9615.ENSCAFP00000001248	Parvin, gamma
1	Red	88	ADAMDEC1	9615.ENSCAFP00000013336	ADAM-like, decysin 1
1	Red	88	SPIC	9615.ENSCAFP00000010400	Spi-C transcription factor (Spi-1/PU.1 related)
1	Red	88	C1QC	9615.ENSCAFP00000021580	Complement component 1, q subcomponent, C chain
1	Red	88	APBB1IP	9615.ENSCAFP00000006427	Amyloid beta (A4) precursor protein-binding, family B, member 1 interacting protein
1	Red	88	GPR65	9615.ENSCAFP00000025551	G protein-coupled receptor 65
1	Red	88	BIRC3	9615.ENSCAFP00000022282	Baculoviral IAP repeat-containing protein 3; Multi-functional protein which regulates not only caspases and apoptosis, but also modulates inflammatory signaling and immunity, mitogenic kinase signaling and cell proliferation, as well as cell invasion and metastasis. Acts as an E3 ubiquitin- protein ligase regulating NF-kappa-B signaling and regulates both canonical and non-canonical NF-kappa-B signaling by acting in opposite directions: acts as a positive regulator of the canonical pathway and suppresses constitutive activation of non-canonical NF-kappa-B signaling.
1	Red	88	COTL1	9615.ENSCAFP00000039200	Coactosin-like 1 (Dictyostelium)
1	Red	88	CD83	9615.ENSCAFP00000041045	CD83 molecule
1	Red	88	REL	9615.ENSCAFP00000004425	V-rel reticuloendotheliosis viral oncogene homolog (avian)
1	Red	88	CTSS	9615.ENSCAFP00000017782	Cathepsin S precursor ; Thiol protease. Key protease responsible for the removal of the invariant chain from MHC class II molecules. The bond- specificity of this proteinase is in part similar to the specificities of cathepsin L and cathepsin N.
1	Red	88	FERMT3	9615.ENSCAFP00000021567	Fermitin family member 3

1	Red	88	PMAIP1	9615.ENSCAFP00000032282	Phorbol-12-myristate-13-acetate-induced protein 1 ; Promotes activation of caspases and apoptosis. Promotes mitochondrial membrane changes and efflux of apoptogenic proteins from the mitochondria. Contributes to p53/TP53-dependent apoptosis after radiation exposure. Promotes proteasomal degradation of MCL1. Competes with BAK1 and with BIM/BCL2L11 for binding to MCL1; can displace BAK1 and BIM/BCL2L11 from their binding sites.
1	Red	88	IL18	9615.ENSCAFP00000038185	Interleukin-18 precursor ; Augments natural killer cell activity in spleen cells and stimulates interferon gamma production in T-helper type I cells
1	Red	88	CD69	9615.ENSCAFP00000020010	CD69 molecule
1	Red	88	TNFRSF14	9615.ENSCAFP00000028709	Tumor necrosis factor receptor superfamily, member 14
1	Red	88	CD74	9615.ENSCAFP00000026761	CD74 molecule, major histocompatibility complex, class II invariant chain
1	Red	88	CASP4	9615.ENSCAFP00000021900	Caspase-1 ; Thiol protease that cleaves IL-1 beta between an Asp and an Ala, releasing the mature cytokine which is involved in a variety of inflammatory processes. Important for defense against pathogens. Cleaves and activates sterol regulatory element binding proteins (SREBPs). Can also promote apoptosis.
1	Red	88	MARCO	9615.ENSCAFP00000007326	Macrophage receptor with collagenous structure
1	Red	88	SKAP1	9615.ENSCAFP00000024759	Src kinase associated phosphoprotein 1
1	Red	88	FAM49A	9615.ENSCAFP00000005546	Family with sequence similarity 49, member A
1	Red	88	P2RY10	9615.ENSCAFP00000025475	Purinergic receptor P2Y, G-protein coupled, 10
1	Red	88	C1QB	9615.ENSCAFP00000039386	Complement component 1, q subcomponent, B chain
1	Red	88	LYZ	9615.ENSCAFP00000000619	Lysozyme C, spleen isozyme ; Lysozymes have primarily a bacteriolytic function; those in tissues and body fluids are associated with the monocyte- macrophage system and enhance the activity of immunoagents
1	Red	88	STK17B	9615.ENSCAFP00000015677	Serine/threonine kinase 17b
1	Red	88	CXCL10	9615.ENSCAFP00000032015	C-X-C motif chemokine 10 ; Chemotactic for monocytes and T-lymphocytes. Binds to CXCR3 (By similarity)
1	Red	88	PRKD2	9615.ENSCAFP00000006334	Protein kinase D2
1	Red	88	CARD11	9615.ENSCAFP00000023996	Caspase recruitment domain family, member 11
1	Red	88	DLA-DRA	9615.ENSCAFP00000001147	MHC class II DR alpha chain precursor
1	Red	88	SKA3**	9615.ENSCAFP00000036892	Spindle and kinetochore associated complex subunit 3; Ska3 promotes chromosome association of the anaphase-promoting complex.
1	Red	88	PARP12	9615.ENSCAFP00000005924	Poly (ADP-ribose) polymerase family, member 12
1	Red	88	BCL2A1	9615.ENSCAFP00000020596	BCL2-related protein A1
1	Red	88	LRRC46	9615.ENSCAFP00000042601	Leucine rich repeat containing 46
1	Red	88	CIITA	9615.ENSCAFP00000027952	Class II, major histocompatibility complex, transactivator
1	Red	88	CCR5	9615.ENSCAFP00000020323	C-C chemokine receptor type 5 ; Receptor for a number of inflammatory CC-chemokines including MIP-1-alpha, MIP-1-beta and RANTES and subsequently transduces a signal by increasing the intracellular calcium ion level. May play a role in the control of granulocytic lineage proliferation or differentiation.
1	Red	88	MAP4K2	9615.ENSCAFP00000020961	Mitogen-activated protein kinase kinase kinase kinase 2
1	Red	88	IFIH1	9615.ENSCAFP00000015359	Interferon induced with helicase C domain 1

1	Red	88	EBI3	9615.ENSCAFP00000028203	Epstein-Barr virus induced 3
1	Red	88	MST4	9615.ENSCAFP00000027770	Uncharacterized protein
1	Red	88	LAPTM5	9615.ENSCAFP00000016550	Lysosomal protein transmembrane 5
1	Red	88	HLA-DRB1	9615.ENSCAFP00000001156	DLA class II histocompatibility antigen, DR-1 beta chain precursor
1	Red	88	ESCO2*	9615.ENSCAFP00000012307	Establishment of cohesion 1 homolog 2 (S. cerevisiae)
1	Red	88	LAIR1	9615.ENSCAFP00000003873	Leukocyte-associated immunoglobulin-like receptor 1
1	Red	88	KCNA2	9615.ENSCAFP00000029266	Potassium voltage-gated channel subfamily A member 2 ; Voltage-gated potassium channel that mediates transmembrane potassium transport in excitable membranes, primarily in the brain and the central nervous system, but also in the cardiovascular system. Prevents aberrant action potential firing and regulates neuronal output. Forms tetrameric potassium-selective channels through which potassium ions pass in accordance with their electrochemical gradient. The channel alternates between opened and closed conformations in response to the voltage difference across the Membrane.
1	Red	88	CKAP2L	9615.ENSCAFP00000032225	Cytoskeleton associated protein 2-like
1	Red	88	MAP4K1	9615.ENSCAFP00000008819	Mitogen-activated protein kinase kinase kinase kinase 1
1	Red	88	KCNA6	9615.ENSCAFP00000040687	Uncharacterized protein
1	Red	88	ITGA4	9615.ENSCAFP00000020903	Integrin, alpha 4 (antigen CD49D, alpha 4 subunit of VLA-4 receptor)
1	Red	88	TRIM59	9615.ENSCAFP00000042145	Tripartite motif containing 59
1	Red	88	CD48	9615.ENSCAFP00000040985	CD48 molecule
1	Red	88	HCLS1	9615.ENSCAFP00000017015	Hematopoietic cell-specific Lyn substrate 1
1	Red	88	GZMK	9615.ENSCAFP00000027147	Granzyme K (granzyme 3; tryptase II)
1	Red	88	PLCB2	9615.ENSCAFP00000013316	Phospholipase C, beta 2
1	Red	88	CCL19	9615.ENSCAFP00000002873	C-C motif chemokine 19 precursor
1	Red	88	LTB	9615.ENSCAFP00000039920	Lymphotoxin-beta
1	Red	88	GPR18	9615.ENSCAFP00000035502	G protein-coupled receptor 18
1	Red	88	TF	9615.ENSCAFP00000009883	Transferrin
1	Red	88	DTX3L	9615.ENSCAFP00000017579	Deltex 3-like (Drosophila)
1	Red	88	FASLG	9615.ENSCAFP00000021643	Uncharacterized protein
1	Red	88	CCL5	9615.ENSCAFP00000026835	C-C motif chemokine 5 precursor ; Chemoattractant for blood monocytes, memory T-helper cells and eosinophils. Causes the release of histamine from basophils and activates eosinophils. May activate several chemokine receptors including CCR1, CCR3, CCR4 and CCR5. May also be an agonist of the G protein-coupled receptor GPR75. Together with GPR75, may play a role in neuron survival through activation of a downstream signaling pathway involving the PI3, Akt and MAP kinases. By activating GPR75 may also play a role in insulin secretion by islet cells
1	Red	88	SLA	9615.ENSCAFP00000001590	Src-like-adaptor

1	Red	88	LRRC8D	9615.ENSCAFP00000029954	Leucine rich repeat containing 8 family, member D
1	Red	88	CD53	9615.ENSCAFP00000029263	CD53 molecule
1	Red	88	LCP1	9615.ENSCAFP00000035137	Lymphocyte cytosolic protein 1 (L-plastin)
1	Red	88	SH2D1A	9615.ENSCAFP00000041409	SH2 domain containing 1A
1	Red	88	SELL	9615.ENSCAFP00000022356	Selectin L; Cell surface adhesion protein. Mediates the adherence of lymphocytes to endothelial cells of high endothelial venules in peripheral lymph nodes. Promotes initial tethering and rolling of leukocytes in endothelia
1	Red	88	ITGAX	9615.ENSCAFP00000024841	Integrin, alpha X (complement component 3 receptor 4 subunit)
1	Red	88	ENPP3	9615.ENSCAFP00000000528	Ectonucleotide pyrophosphatase/phosphodiesterase 3
1	Red	88	HS3ST3A1	9615.ENSCAFP00000026405	Heparan sulfate (glucosamine) 3-O-sulfotransferase 3A1
1	Red	88	NIPA1	9615.ENSCAFP00000014331	Non imprinted in Prader-Willi/Angelman syndrome 1
1	Red	88	CD2	9615.ENSCAFP00000014445	CD2 molecule
1	Red	88	FYB	9615.ENSCAFP00000035897	FYN binding protein
1	Red	88	CAMK1D	9615.ENSCAFP00000007134	Calcium/calmodulin-dependent protein kinase ID
1	Red	88	ARPC4	9615.ENSCAFP00000007935	Actin related protein 2/3 complex, subunit 4, 20kDa
1	Red	88	IDO1	9615.ENSCAFP00000008594	Indoleamine 2,3-dioxygenase 1
1	Red	88	MXD3	9615.ENSCAFP00000024190	MAX dimerization protein 3
1	Red	88	CASP12	9615.ENSCAFP00000038116	Caspase-12 ; Involved in the activation cascade of caspases responsible for apoptosis execution
2	Yellow	37	CCNF*	9615.ENSCAFP00000028622	Cyclin F
	Yellow	37	AURKB*	9615.ENSCAFP00000025043	Aurora kinase B
2	Yellow	37	CDCA3*	9615.ENSCAFP00000021602	F-box-like protein which is required for entry into mitosis
2	Yellow	37	CCNA2*	9615.ENSCAFP00000006129	Cyclin A2
2	Yellow	37	CENPL**	9615.ENSCAFP00000021422	Centromere protein L
2	Yellow	37	TUBB	9615.ENSCAFP00000000649	Tubulin, beta class I; Tubulin is the major constituent of microtubules. It binds two moles of GTP, one at an exchangeable site on the beta chain and one at a non-exchangeable site on the alpha chain
2	Yellow	37	EZH2	9615.ENSCAFP00000005090	Enhancer of zeste homolog 2 (Drosophila)
2	Yellow	37	KIF15*?	9615.ENSCAFP00000020805	Kinesin family member 15
2	Yellow	37	HMGB2	9615.ENSCAFP00000011606	High mobility group box 2
2	Yellow	37	CEP152**	9615.ENSCAFP00000038873	Centrosomal protein 152kDa
2	Yellow	37	PTTG1*	9615.ENSCAFP00000043220	Pituitary tumor-transforming 1
2	Yellow	37	NUF2**	9615.ENSCAFP00000019550	NUF2, NDC80 kinetochore complex component, homolog (S. cerevisiae)

2	Yellow	37	OAS1	9615.ENSCAFP00000013118	2'-5'-oligoadenylate synthase 1
2	Yellow	37	SPC25**	9615.ENSCAFP00000017454	SPC25, NDC80 kinetochore complex component, homolog (S. cerevisiae)
2	Yellow	37	PSMB10	9615.ENSCAFP00000030153	Proteasome (prosome, macropain) subunit, beta type, 10; The proteasome is a multicatalytic proteinase complex which is characterized by its ability to cleave peptides with Arg, Phe, Tyr, Leu, and Glu adjacent to the leaving group at neutral or slightly basic pH. The proteasome has an ATP-dependent proteolytic activity
2	Yellow	37	VRK1	9615.ENSCAFP00000026262	Vaccinia related kinase 1
2	Yellow	37	CDC20*	9615.ENSCAFP00000007875	Cell division cycle 20 homolog (S. cerevisiae)
2	Yellow	37	NEK2*/**	9615.ENSCAFP00000032514	NIMA-related kinase 2
2	Yellow	37	TUBA1B	9615.ENSCAFP00000012688	Tubulin, alpha 1b; Tubulin is the major constituent of microtubules. It binds two moles of GTP, one at an exchangeable site on the beta chain and one at a non-exchangeable site on the alpha chain
2	Yellow	37	INCENP**	9615.ENSCAFP00000023376	Inner centromere protein antigens 135/155kDa
2	Yellow	37	CCNB2*	9615.ENSCAFP00000024416	Cyclin B2
2	Yellow	37	MLF1IP**	9615.ENSCAFP00000011465	MLF1 interacting protein; also called CENPU, required for centromere assembly
2	Yellow	37	CASP8	9615.ENSCAFP00000017899	Caspase-8
2	Yellow	37	SGOL1*/**	9615.ENSCAFP00000008681	Shugoshin-like 1 (S. pombe)
2	Yellow	37	PSMB8	9615.ENSCAFP00000001185	Proteasome subunit beta type-8 ; The proteasome is a multicatalytic proteinase complex which is characterized by its ability to cleave peptides with Arg, Phe, Tyr, Leu, and Glu adjacent to the leaving group at neutral or slightly basic pH. The proteasome has an ATP-dependent proteolytic activity. This subunit is involved in antigen processing to generate class I binding peptides (By similarity). Required for adipocyte differentiation.
2	Yellow	37	PSME2	9615.ENSCAFP00000017612	Proteasome (prosome, macropain) activator subunit 2 (PA28 beta)
2	Yellow	37	BUB1**	9615.ENSCAFP00000037890	Budding uninhibited by benzimidazoles 1 homolog (yeast)
2	Yellow	37	SPC24**	9615.ENSCAFP00000025835	SPC24, NDC80 kinetochore complex component, homolog (S. cerevisiae)
2	Yellow	37	CDKN3	9615.ENSCAFP00000021952	Cyclin-dependent kinase inhibitor 3; May play a role in cell cycle regulation. Dual specificity phosphatase active toward substrates containing either phosphotyrosine or phosphoserine residues
2	Yellow	37	PLK1*	9615.ENSCAFP00000039127	Polo-like kinase 1
2	Yellow	37	CASC5**	9615.ENSCAFP00000013547	Cancer susceptibility candidate 5
2	Yellow	37	UBE2C*	9615.ENSCAFP00000014322	Ubiquitin-conjugating enzyme E2C
2	Yellow	37	NDC80*/**	9615.ENSCAFP00000027183	NDC80 kinetochore complex component homolog (S. cerevisiae)
2	Yellow	37	DLGAP5*	9615.ENSCAFP00000022150	Potential cell cycle regulator that may play a role in carcinogenesis of cancer cells. Mitotic phosphoprotein regulated by the ubiquitin-proteasome pathway. Also called HURP.
2	Yellow	37	LRR1	9615.ENSCAFP00000020889	Leucine rich repeat protein 1
2	Yellow	37	NUSAP1*	9615.ENSCAFP00000014023	Nucleolar and spindle associated protein 1
2	Yellow	37	CDK1	9615.ENSCAFP00000019027	Cyclin-dependent kinase 1
3	Green	21	NCAPD2***	9615.ENSCAFP00000022300	non-SMC condensin I complex, subunit D2; Regulatory subunit of the condensin complex, a complex required for conversion of interphase chromatin into mitotic-like condense chromosomes. The condensin complex probably introduces positive supercoils into relaxed DNA in the presence of type I topoisomerases and converts nicked DNA into positive

					knotted forms in the presence of type II topoisomerases
3	Green	21	ISG20	9615.ENSCAFP00000016938	Interferon stimulated exonuclease gene 20kDa
	Green	21	NCAPH***	9615.ENSCAFP00000009828	non-SMC condensin I complex, subunit H; Regulatory subunit of the condensin complex, a complex required for conversion of interphase chromatin into mitotic-like condense chromosomes
3	Green	21	E2F1	9615.ENSCAFP00000011055	E2F transcription factor 1
3	Green	21	ENO2	9615.ENSCAFP00000021430	Enolase 2 (gamma, neuronal)
3	Green	21	PFKP	9615.ENSCAFP00000008224	Phosphofructokinase, platelet; Catalyzes the phosphorylation of D-fructose 6-phosphate to fructose 1,6-bisphosphate by ATP, the first committing step of glycolysis
3	Green	21	NCAPG*/***	9615.ENSCAFP00000024530	non-SMC condensin I complex, subunit G
3	Green	21	AK8	9615.ENSCAFP00000029454	Adenylate kinase 8
3	Green	21	ACTR3B	9615.ENSCAFP00000037793	ARP3 actin-related protein 3 homolog B (yeast)
3	Green	21	RAD51	9615.ENSCAFP00000013557	DNA repair protein RAD51 homolog 1 ; Participates in a common DNA damage response pathway associated with the activation of homologous recombination and double-strand break repair. Binds to single and double-stranded DNA and exhibits DNA-dependent ATPase activity. Underwinds duplex DNA and forms helical nucleoprotein filaments. Part of a PALB2- scaffolded HR complex containing BRCA2 and RAD51C and which is thought to play a role in DNA repair by HR. Plays a role in regulating mitochondrial DNA copy number under conditions of oxidative stress in the presence of RAD51C and XRCC3
3	Green	21	PRPSAP2	9615.ENSCAFP00000026958	Phosphoribosyl pyrophosphate synthetase-associated protein 2
3	Green	21	DCP2	9615.ENSCAFP00000037100	DCP2 decapping enzyme homolog (S. cerevisiae)
3	Green	21	KIAA0101	9615.ENSCAFP00000040037	also called PCLAF; PCNA-binding protein that acts as a regulator of DNA repair during DNA replication
3	Green	21	WRN	9615.ENSCAFP00000032498	Werner syndrome, RecQ helicase-like
3	Green	21	SPAG5**	9615.ENSCAFP00000027622	Sperm associated antigen 5; interacts with Ndc80 and CEP55
3	Green	21	ASPM**	9615.ENSCAFP00000016776	Abnormal spindle-like microcephaly-associated protein homolog ; Probable role in mitotic spindle regulation and coordination of mitotic processes. May have a preferential role in regulating neurogenesis.
3	Green	21	TAP2	9615.ENSCAFP00000001183	ATP Binding Cassette Subfamily B Member; also called ABCB3
3	Green	21	KCNAB2	9615.ENSCAFP00000028869	Potassium voltage-gated channel, shaker-related subfamily, beta member 2
3	Green	21	ESPL1*	9615.ENSCAFP00000010445	Extra spindle pole bodies homolog 1 (S. cerevisiae)
3	Green	21	ACP5	9615.ENSCAFP00000025519	Acid phosphatase type 5; Uncharacterized protein
3	Green	21	TOP2A	9615.ENSCAFP00000042966	Topoisomerase (DNA) II alpha 170kDa; Control of topological states of DNA by transient breakage and subsequent rejoining of DNA strands. Topoisomerase II makes double-strand breaks.
4	Medium Aqua Marine	16	ARHGDIB	9615.ENSCAFP00000018985	Rho GDP dissociation inhibitor (GDI) beta
4	Medium Aqua Marine	16	ARHGAP30	9615.ENSCAFP00000018695	Rho GTPase activating protein 30

4	Medium Aqua Marine	16	HMHA1	9615.ENSCAFP00000031556	Histocompatibility (minor) HA-1
	Medium Aqua Marine	16	FGD3	9615.ENSCAFP00000003318	FYVE, RhoGEF and PH domain containing 3
4	Medium Aqua Marine	16	ECT2	9615.ENSCAFP00000037855	Epithelial cell transforming sequence 2 oncogene
4	Medium Aqua Marine	16	ARHGAP15	9615.ENSCAFP00000008184	Rho GTPase activating protein 15
4	Medium Aqua Marine	16	PREX1	9615.ENSCAFP00000036141	Phosphatidylinositol-3,4,5-trisphosphate-dependent Rac exchange factor 1
4	Medium Aqua Marine	16	PRKCB	9615.ENSCAFP00000025992	Protein kinase C, beta
4	Medium Aqua Marine	16	GMIP	9615.ENSCAFP00000020882	GEM interacting protein
4	Medium Aqua Marine	16	ARHGAP9	9615.ENSCAFP00000000325	Rho GTPase activating protein 9
4	Medium Aqua Marine	16	MX2	9615.ENSCAFP00000014935	Interferon-induced GTP-binding protein Mx2 ; Interferon-induced dynamin-like GTPase with antiviral activity against vesicular stomatitis virus (VSV)
4	Medium Aqua Marine	16	PSD4	9615.ENSCAFP00000010829	Pleckstrin and Sec7 domain containing 4
4	Medium Aqua Marine	16	RACGAP1	9615.ENSCAFP00000012284	Rac GTPase activating protein 1
4	Medium Aqua Marine	16	TAGAP	9615.ENSCAFP00000038236	T-cell activation RhoGTPase activating protein
4	Medium Aqua Marine	16	RHOH	9615.ENSCAFP00000023421	Ras homolog family member H
4	Medium Aqua Marine	16	FAM13B	9615.ENSCAFP00000001644	Family with sequence similarity 13, member B
5	Dark Cyan	16	CYBA	9615.ENSCAFP00000029449	Cytochrome b-245, alpha polypeptide ; Critical component of the membrane-bound oxidase of phagocytes that generates superoxide. Associates with NOX3 to form a functional NADPH oxidase constitutively generating superoxide
5	Dark Cyan	16	FGR	9615.ENSCAFP00000017792	Gardner-Rasheed feline sarcoma viral (v-fgr) oncogene homolog
5	Dark Cyan	16	CD3D	9615.ENSCAFP00000038117	CD3d molecule, delta (CD3-TCR complex)

5	Dark Cyan	16	ITGAL	9615.ENSCAFP00000024419	Integrin, alpha L (antigen CD11A (p180), lymphocyte function-associated antigen 1; alpha polypeptide)
5	Dark Cyan	16	PTPN6	9615.ENSCAFP00000021345	Protein tyrosine phosphatase, non-receptor type 6
5	Dark Cyan	16	NCF4	9615.ENSCAFP00000002195	Neutrophil cytosolic factor 4, 40kDa
5	Dark Cyan	16	DAPP1	9615.ENSCAFP00000015548	Dual adaptor of phosphotyrosine and 3-phosphoinositides
5	Dark Cyan	16	NCKAP1L	9615.ENSCAFP00000009656	NCK-associated protein 1-like
5	Dark Cyan	16	ITGB2	9615.ENSCAFP00000016262	Integrin beta
5	Dark Cyan	16	CD80	9615.ENSCAFP00000016197	T-lymphocyte activation antigen CD80 precursor
5	Dark Cyan	16	CD86	9615.ENSCAFP00000017296	T-lymphocyte activation antigen CD86 precursor
5	Dark Cyan	16	PIK3CD	9615.ENSCAFP00000029210	Phosphatidylinositol-4,5-bisphosphate 3-kinase, catalytic subunit delta
5	Dark Cyan	16	CYFIP2	9615.ENSCAFP00000025820	Cytoplasmic FMR1 interacting protein 2
5	Dark Cyan	16	CYBB	9615.ENSCAFP00000020532	Cytochrome b-245, beta polypeptide
5	Dark Cyan	16	DLA-64	9615.ENSCAFP00000031410	MHC class I DLA-64 precursor ; Involved in the presentation of foreign antigens to the immune system
5	Dark Cyan	16	VAV1	9615.ENSCAFP00000027443	Vav 1 guanine nucleotide exchange factor
6	Purple	8	MCM5	9615.ENSCAFP00000002480	Minichromosome maintenance complex component 5
6	Purple	8	FEN1	9615.ENSCAFP00000043067	Flap structure-specific endonuclease 1; Structure-specific nuclease with 5'-flap endonuclease and 5'-3' exonuclease activities involved in DNA replication and repair. During DNA replication, cleaves the 5'-overhanging flap structure that is generated by displacement synthesis when DNA polymerase encounters the 5'-end of a downstream Okazaki fragment. It enters the flap from the 5'-end and then tracks to cleave the flap base, leaving a nick for ligation.
6	Purple	8	RAD18	9615.ENSCAFP00000008290	RAD18 homolog (S. cerevisiae)
	Purple	8	KIF4A*	9615.ENSCAFP00000024762	Uncharacterized protein
6	Purple	8	MCM6	9615.ENSCAFP00000007692	Minichromosome maintenance complex component 6
6	Purple	8	MCM10	9615.ENSCAFP00000007092	Minichromosome maintenance complex component 10
6	Purple	8	KIF23*	9615.ENSCAFP00000025844	Kinesin family member 23
6	Purple	8	GINS4	9615.ENSCAFP00000008567	GINS complex subunit 4 (Sld5 homolog); The GINS complex plays an essential role in the initiation of DNA replication

* APC substrates

** Kinetochore and Spindle Assembly Checkpoint associated proteins

*** Components of the chromosome condensin complex

Supplemental Table S5

Canine 4 tumor up 3 FC:remission down 2 FC:no remission up 3 FC (27 genes)

Red	CKAP2L*/**	Cytoskeleton associated protein 2-like
Red	CDC20*	Cell division cycle 20 homolog (S. cerevisiae)
Red	NEK2*/**	NIMA-related kinase 2
Red	PTTG1*	Pituitary tumor-transforming 1
Red	ECT2	Epithelial cell transforming sequence 2 oncogene
Red	CCNB2*	Cyclin B2
Red	NUF2**	NUF2, NDC80 kinetochore complex component, homolog (S. cerevisiae)
Red	UBE2C*	Ubiquitin-conjugating enzyme E2C
Red	DLGAP5*	Discs, large (Drosophila) homolog-associated protein 5
Red	CDCA3*	F-box-like protein which is required for entry into mitosis
Red	CCNA2*	Cyclin A2
Green	C4BPA	Complement component 4 binding protein, alpha
Green	CXCL10	C-X-C motif chemokine 10 ; Chemotactic for monocytes and T-lymphocytes. Binds to CXCR3 (By similarity)
Green	MCM5	Minichromosome maintenance complex component 5
Green	KIF15*?	Kinesin family member 15
Green	APOC1	Apolipoprotein C-I Truncated apolipoprotein C-I; Inhibitor of lipoprotein binding to the low density lipoprotein (LDL) receptor, LDL receptor-related protein, and very low density lipoprotein (VLDL) receptor. Associates with high density lipoproteins (HDL) and the triacylglycerol-rich lipoproteins in the plasma and makes up about 10% of the protein of the VLDL and 2% of that of HDL. Appears to interfere directly with fatty acid uptake and is also the major plasma inhibitor of cholesteryl ester transfer protein (CETP). Binds free fatty acids and reduces their intracellular esterificatio [...]
Green	MIS18A**	MIS18 kinetochore protein homolog A (S. pombe)
Green	HIST1H1A	Histone cluster 1, H1a
Green	MCM10	Minichromosome maintenance complex component 10
Green	CD70	CD70 molecule
Dark Cyan	SPAG5**	Sperm associated antigen 5
Dark Cyan	HIST1H2AC	Histone cluster 1, H2ac
Dark Cyan	TOP2A	Topoisomerase (DNA) II alpha 170kDa; Control of topological states of DNA by transient breakage and subsequent rejoining of DNA strands. Topoisomerase II makes double-strand breaks
Dark Cyan	RAD51	DNA repair protein RAD51 homolog 1 ; Participates in a common DNA damage response pathway associated with the activation of homologous recombination and double-strand break repair. Binds to single and double-stranded DNA and exhibits DNA-dependent ATPase activity. Underwinds duplex DNA and forms helical nucleoprotein filaments. Part of a PALB2- scaffolded HR complex containing BRCA2 and RAD51C and which is thought to play a role in DNA repair by HR. Plays a role in regulating mitochondrial DNA copy number under conditions of oxidative stress in the presence of RAD51C and XRCC3
Dark Cyan	NCAPG*/***	non-SMC condensin I complex, subunit G

*	APC substrates
**	Kinetochore and Spindle Assembly Checkpoint associated proteins
***	Components of the chromosome condensin complex
